# Effect of Acylated and Nonacylated Anthocyanins on Urine Metabolic Profile during the Development of Type 2 Diabetes in Zucker Diabetic Fatty Rats

**DOI:** 10.1021/acs.jafc.2c06802

**Published:** 2022-11-21

**Authors:** Kang Chen, Xuetao Wei, Jian Zhang, Maaria Kortesniemi, Yumei Zhang, Baoru Yang

**Affiliations:** † Food Sciences, Department of Life Technologies, University of Turku, FI-20014 Turu, Finland; ‡ Beijing Key Laboratory of Toxicological Research and Risk Assessment for Food Safety, Department of Toxicology, School of Public Health, Peking University, Beijing 100191, China; § Department of Nutrition and Food Hygiene, School of Public Health, Peking University, Beijing 100191, China

**Keywords:** acylated anthocyanins, nonacylated anthocyanins, ^1^H NMR metabolomics, urine, type 2 diabetes, Zucker diabetic fatty rat, purple potato, bilberry

## Abstract

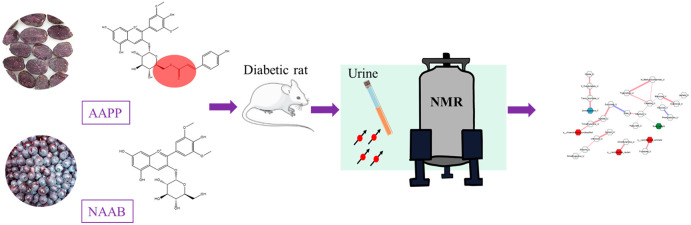

The effect of nonacylated and acylated anthocyanins on urinary metabolites in diabetic rats was investigated. Nonacylated anthocyanins extract from bilberries (NAAB) or acylated anthocyanins extract from purple potatoes (AAPP) was given to Zucker diabetic fatty (ZDF) rats for 8 weeks at daily doses of 25 and 50 mg/kg body weight. ^1^H NMR metabolomics was applied to study alterations in urinary metabolites from three time points (weeks 1, 4, and 8). Both types of anthocyanins modulated the metabolites associated with the tricarboxylic acid cycle, gut microbiota metabolism, and renal function at weeks 1 and 4, such as 2-oxoglutarate, fumarate, alanine, trigonelline, and hippurate. In addition, only a high dose of AAPP decreased monosaccharides, formate, lactate, and glucose levels at week 4, suggesting improvement in energy production in mitochondria, glucose homeostasis, and oxidative stress. This study suggested different impacts of AAPP and NAAB on the metabolic profile of urine in diabetes.

## Introduction

Type 2 diabetes (T2D) is an increasing threat to public health globally. It is predicted that over 693 million people will be affected by type 2 diabetes worldwide by 2045.^1^ Anthocyanins, a class of polyphenols, are abundant in colored fruits and vegetables.^2^ With or without the acylation of the glucoside, anthocyanins can be classified into nonacylated anthocyanins and acylated anthocyanins, respectively. Acylated anthocyanins have been observed to have higher stability^3^ and antioxidant activities^4^ than their nonacylated counterparts. Antidiabetic activities of anthocyanins mainly from berries have been widely studied,^5^ where their antioxidant and anti-inflammation activities play a crucial role. Our previous studies have shown that bilberry nonacylated anthocyanins and purple potato acylated anthocyanins affected the plasma and hepatic metabolic profile, hepatic transcriptome, gut metabolic profile, and gut microbiota differently, with acylated anthocyanins showing more beneficial effects.^6−8^ However, the impact of dietary supplementation with anthocyanin extract on urinary metabolites in the development of diabetes has not been reported. Urine, as a sterile and easy-to-obtain biofluid, contains endogenous waste metabolites, metabolic breakdown products from drugs and foods, and metabolites from bacteria.^9^ In addition, the urine metabolic profile constitutes other information that pertains to both renal function and metabolic wastes, which is important to evaluate the effect of different types of anthocyanins on T2D. A difference between urine and other biofluids is that urine is not homeostatic. Thus, the urinary metabolites could show more significant changes when receiving different interventions.

Urine ^1^H NMR metabolomics has been broadly used to identify biomarkers in T2D and the metabolic changes of the administration of polyphenols-rich diets. Urine ^1^H NMR metabolomics has revealed thirty-three urinary metabolites to be significantly altered in diabetic mice, represented by increased metabolites associated with the tricarboxylic acid (TCA) cycle, monosaccharides, and others including dimethylglycine and trigonelline, etc.^10^ Decreased urinary levels of hippurate, allantoin, creatinine, taurine, and α-ketoglutarate have been observed in diabetic ZDF (Zucker diabetic fatty) rats with ^1^H NMR metabolomics.^11^ Polyphenols-rich diets have shown modulatory effects on urinary metabolites. Intake of cranberry juice has been reported to increase urinary hippurate level.^12^ Berry mixture consumption has elevated urinary proline in rats under a low-salt diet.^13^ Cranberry procyanidins’ consumption has increased the content of succinate, lactate, and hippurate and decreased citrate and α-ketoglutarate in the urine of rats.^14^

The advantages of using ^1^H NMR metabolomics are the minimal requirement for sample preparation, the robust and reproducible measurements, and the nondestructive nature of the analysis.^15^ In this study, ^1^H NMR-based metabolomics combined with multivariate and univariate statistics were applied to compare the effects of nonacylated anthocyanins extracted from bilberries (NAAB) and acylated anthocyanins extracted from purple potatoes (AAPP) on urinary metabolites in ZDF rats at three time points during the intervention and development of diabetes.

## Materials and Methods

### Animals and Diets

In this study, a T2D model was induced by feeding ZDF (*fa/fa*) rats with a high-fat diet. ZDF rat is a spontaneous genetic diabetes model with a *leptin* receptor gene defect caused by a missense mutation.^16^ Lean Zucker rats (*fa/+*) were chosen as healthy controls. Anthocyanins were extracted from tubers of a Finnish variety of purple potato (*Solanum tuberosum* L. “Synkeä Sakari”) and bilberries (*Vaccinium myrtillus* L.). Identification and quantification of both anthocyanin extracts were described previously.^6^ 98.97% of anthocyanins in AAPP were acylated, and all anthocyanins in NAAB were nonacylated.^6^ The anthocyanins of NAAB consisted of mostly galactosides, arabinosides, and glucosides of cyanidin, petunidin, delphinidin, malvidin, and peonidin.^6^ The AAPP consisted of acylated anthocyanins, dominated by petunidin-coumaroyl-rutinoside-glucoside followed by petunidin-caffeoyl-rutinoside-glucoside and peonidin-coumaroyl-rutinoside-glucoside.^6^

Group designation, the composition of diets, and dosage justification were described in our previous publication.^6^ In short, 40 ZDF rats were divided into five groups: ZDF rats fed with a high-fat diet (diabetic model, the M group); ZDF rats fed with a high-fat diet and gavaged with a low dose of bilberry anthocyanins extract (25 mg/kg body weight/day, the L-NAAB group); ZDF rats fed with high-fat diet and gavaged with a low dose of potato anthocyanins extract (25 mg/kg body weight/day, the L-AAPP group); ZDF rats fed with a high-fat diet and gavaged with a high dose of bilberry anthocyanins extract (50 mg anthocyanins/kg body weight/day, the H-NAAB group); ZDF rats fed with a high-fat diet and gavaged with a high dose of potato anthocyanins extract (50 mg/kg body weight/day, the H-AAPP group). The dosages were chosen based on previous animal studies^17−19^ and our previous clinical trial.^20^ The dosages of anthocyanins used in this study would correspond to 10–20 g of dried bilberries and 330–660 g of fresh potatoes per day, which are achievable in daily life.^21,22^

As comparisons, 16 lean Zucker rats were divided into two groups: one fed with a high-fat diet as the control group (Con), and the other with a normal diet (ND). The Institutional Animal Ethics Committee of Peking University granted ethical approval (number LA2016285) for this animal study. The rats were housed in metabolic cages (3700M071, Tecniplast, Italy) for 24 h and had access to feed and water ad libitum; urine was collected during the second to the third day of week 1, week 4, and week 8 of intervention. Sodium azide (1.0% w/v) was added to the collected urine samples. Our previous study has shown the diabetic model group (M) was characterized by higher levels of food intake, water intake, body weight, plasma triglyceride, plasma blood urea nitrogen, plasma total protein, and plasma glucose compared to the lean Zucker rats, indicating the establishment of diabetes in the model group.^6^ All the anthocyanin extracts-treated groups displayed lower levels of plasma glucose compared to the model group without anthocyanin treatments.^6^

### ^1^H NMR Measurements

Urine samples were thawed on ice, and an aliquot of 800 μL was taken from each sample and centrifuged at 3000 for 15 min, after which 400 μL of the supernatant was collected and mixed with 200 μL of phosphate buffer (90 mmol/L NaH_2_PO_4_, pH = 7.4) to reduce pH variations. Thereafter, 60 μL of Chenomx Internal Standard containing 5 mM DSS-*d*_6_ (Edmonton, Alberta, Canada) was added to 560 μL of the mixed sample in an Eppendorf tube and vortexed for 20 s. 600 μL of the resulted solution was transferred to a 5 mm NMR tube. The parameters of ^1^H NMR acquisition were shown in our previous study.^6^ The acquired spectra were aligned using a chemical shift of DSS-*d*_6_ (δ = 0.000 ppm) and binned with 0.001 ppm interval. Binned data were subjected to the *i*coshift program in MATLAB (R2012a, The Mathworks Inc., Natick, MA, USA) to perform spectra alignment. Besides regions of residual water (δ 4.700–4.900) and redundant spectral regions (regions before δ 0.700 and after δ 9.500), regions of glucose (δ 3.210–3.290, δ 3.350–3.575, δ 3.685–3.950, δ 4.550–4.700, and δ 5.200–5.300) and urea (δ 5.520–6.000) were also removed prior to the data normalization to the total area due to the fact that the glucose and urea signals dominating the metabolic change in the spectra of urine from ZDF rats would deteriorate the normalization performance.^23,24^ To quantify the glucose and urea, a median-based probabilistic quotient normalization method was applied to the spectra.^23,24^ Metabolite identification was verified by using Chenomx Profiler 8.6 software (Chenomx Inc., Edmonton, Alberta, Canada) and refs (25 and 26). *J*-resolved spectroscopy (JRES) and ^1^H–^13^C heteronuclear single-quantum correlation spectroscopy (HSQC) were performed to confirm the identifications.

### Statistical Analysis

The number of bins was decreased by adding up the intensities of the consecutive 10 bins. The binned data were Pareto-scaled. PLS-DA models were generated from SIMCA-P+ (V12.0, Umetrics AB, Umeå, Sweden) and validated by permutation test^27^ and cross-validated analysis of variance (CV-ANOVA).^28^ For univariate analysis, a parametric one-way analysis of variance (ANOVA) was performed if data were normally distributed; otherwise, the nonparametric Kruskal–Wallis test was used, and post hoc Dunn’s test or Fisher’s LSD test was applied between groups. The statistical significances are expressed as * or # *p* < 0.05, ** or ## *p* < 0.01, and *** or ### *p* < 0.001. Heat maps and metabolic pathway analysis were generated with the MetaboAnalyst tool (https://www.metaboanalyst.ca/). Correlation analysis of urinary metabolites to plasma metabolites and clinical traits was conducted based on a debiased sparse partial analysis in Cytoscape (Version:3.2.1; https://cytoscape.org/) (*r* > 0.4 or *r* < −0.4; *p* < 0.05). In our previous study, we measured the plasma, hepatic, and fecal metabolites, fecal microbiota, as well as clinical traits,^6−8^ which were used for correlation analysis with the urine metabolites in the current study.

## Results

### Multivariate Analysis of Binned Data from Urine ^1^H NMR Metabolomics

**Figure 1:**
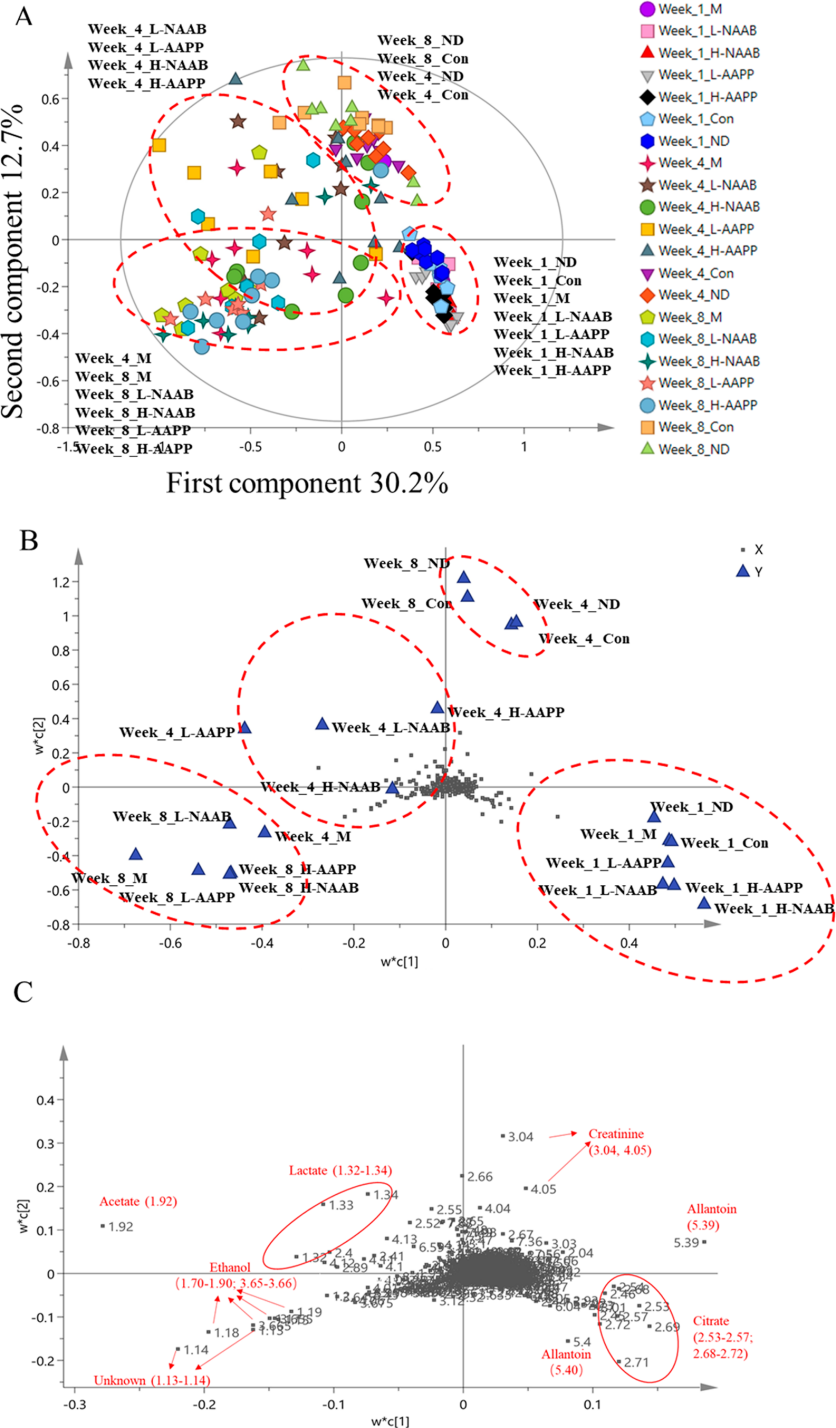
PLS-DA score (A) and loading plot (B) of binned data from ^1^H NMR spectra of urine at three time points. Zoomed-in loading plot (C). Unknown indicates the unknown metabolite identified from bins 1.13–1.14 ppm of ^1^H NMR spectra.

PLS-DA score plots and loading plots were generated based on ^1^H NMR spectra binned data and showed a clear separation and trajectories based on the intervention time points (Figure 1A and B). The first and second components explained 30.2% and 12.7% of the total variance (*R*^2^*X*_(cum)_ = 0.745, *R*^2^*Y*_(cum)_ = 0.247, and *Q*^2^_(cum)_ = 0.104; permutation test *Y*-intercepts: *R*^2^*Y* = 0.125, *Q*^2^*Y* = −0.229; CV-ANOVA*p*-value = 1.12e-18). In the loading plot (Figure 1B), the urine metabolic profiles of Con and ND groups exhibited a trajectory from the fourth quadrant to the first quadrant as the time proceeded from week 1 to weeks 4 and 8; similarly, the M group exhibited a trajectory from the fourth quadrant to the third quadrant as the time proceeded from week 1 to weeks 4 and 8. These results indicated major metabolic changes in the development of diabetes in diabetic rats, and also the growth of lean Zucker rats occurred during the time between week 1 and week 4. Anthocyanin-treated groups, especially the group fed with low-dose nonacylated anthocyanin extract (L-NAAB) and the rats treated with the acylated anthocyanin extract (L-AAPP and H-AAPP), shifted the metabolic profile of diabetic ZDF rats from the fourth quadrant toward the second quadrant and then to the third quadrant as the time proceeded from week 1 to weeks 4 and 8. Next, the loading plot was zoomed in to show the metabolites contributing to the group classification (Figure 1C). The changes of citrate and allantoin contributed to the separation of the samples at week 1 from those collected at weeks 4 and 8. The change of creatinine contributed to the classification of the Con and ND groups at weeks 4 and 8 from other groups. The change of lactate differentiated the urinary metabolic profile of ZDF rats (M, L/H-AAPP, and L/H-NAAB groups) at week 4 from others, whereas acetate, ethanol, and the unknown metabolites (region δ 1.13–1.14 ppm) differentiated the urinary metabolic profile of ZDF rats at week 8 from others.

### Effects of Anthocyanin Extracts on Urinary Metabolites in ZDF Rats

**Figure 2:**
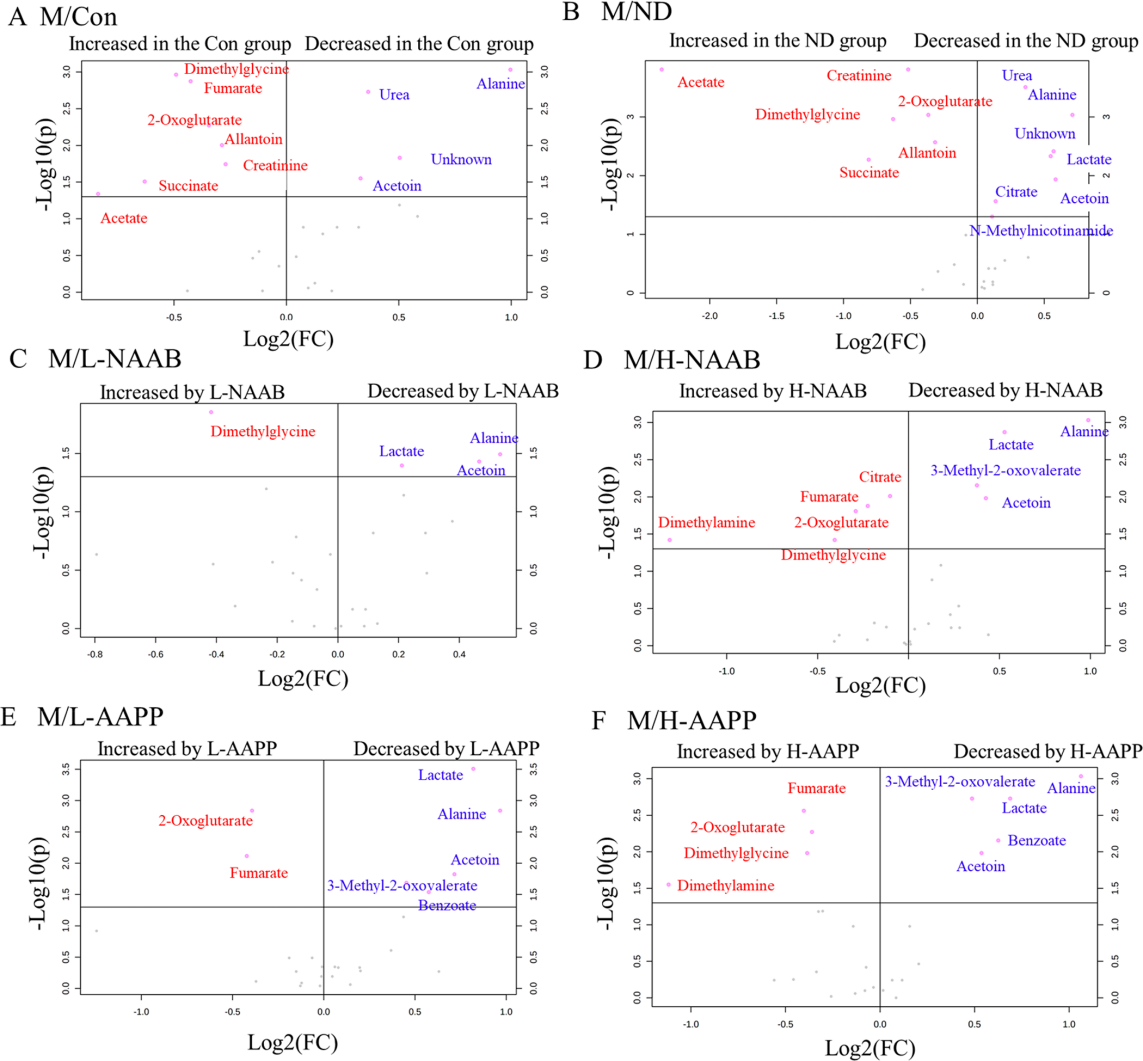
Volcano plots showing the significantly different metabolites in urine at week 1 between groups M/Con (A), M/ND (B), M/L-NAAB (C), M/H-NAAB (D), M/L-AAPP (E), and M/H-AAPP (F). Significance versus log2 fold change is plotted on the y and x axes, respectively. Unknown indicates the unknown metabolite in the 1.13–1.14 ppm region of ^1^H NMR spectra.

**Figure 3:**
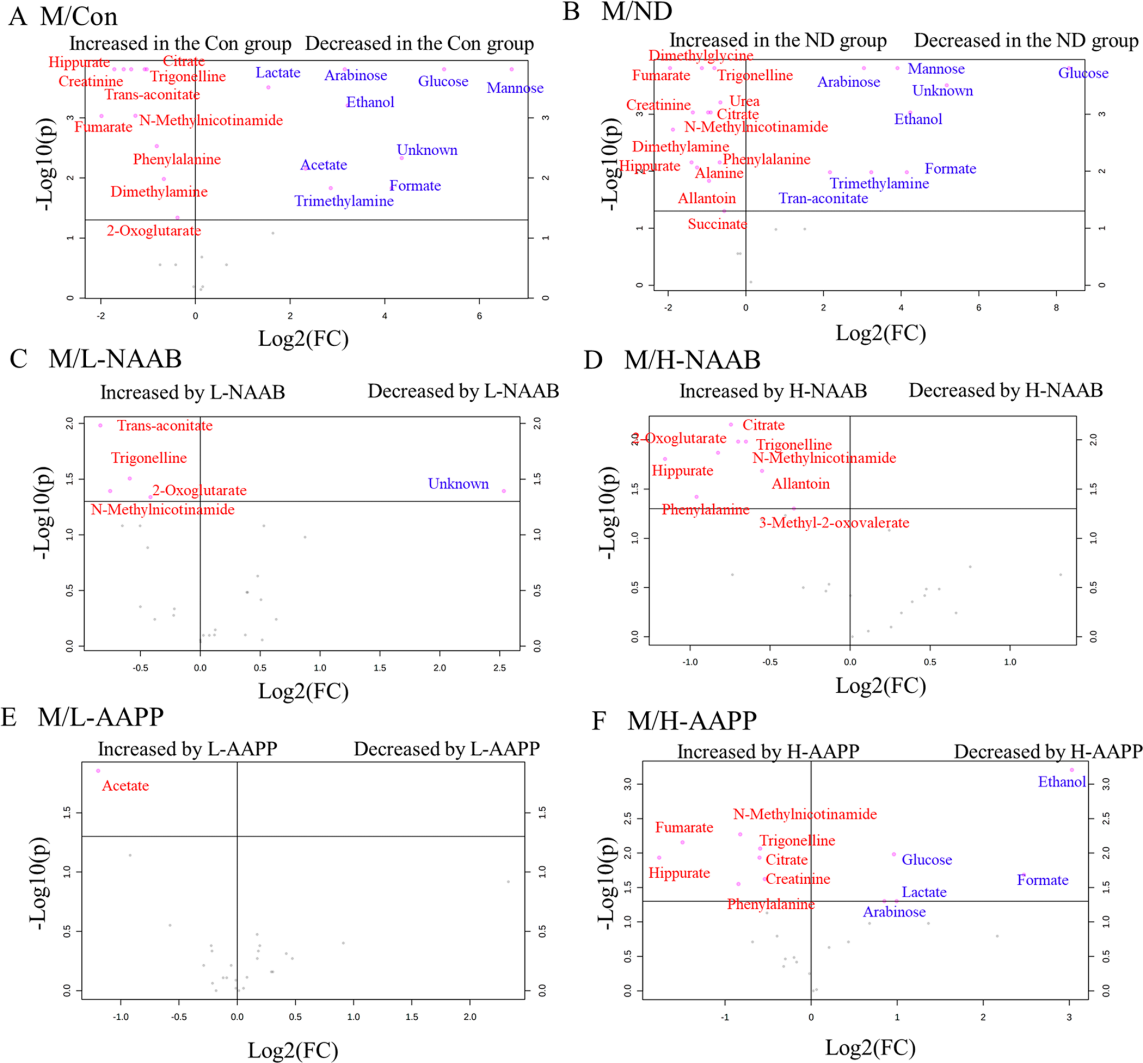
Volcano plots showing the significantly different metabolites in urine at week 4 between groups M/Con (A), M/ND (B), M/L-NAAB (C), M/H-NAAB (D), M/L-AAPP (E), and M/H-AAPP (F). Significance versus log2 fold change is plotted on the y and x axes, respectively. Unknown indicates the unknown metabolite in the 1.13 to 1.14 ppm region of ^1^H NMR spectra.

The representative ^1^H NMR spectrum of the urine sample is presented in Figure S1. A variety of metabolite resonances were revealed in ^1^H NMR spectra, of which 29 metabolites were identified; the chemical shifts and corresponding binned area used for quantification are listed in Table S1. To identify the metabolites that were altered among the groups at each time point, univariate analyses were performed, and significantly changed metabolites (*p* < 0.05) were presented in volcano plots (Figure 2, Figure 3, and Figure S2). Fold changes of each metabolite compared to the M group are shown in Tables S2–S4.

At week 1, the M group showed lower levels of dimethylglycine, creatinine, 2-oxoglutarate, succinate, acetate, and allantoin and increased acetoin, alanine, the unknown metabolite, and urea compared to the Con and ND groups (Figure 2A–B). Both anthocyanin extracts exhibited a modulatory effect on urinary metabolites already after a short period of intervention (2–3 days). At high dose, both anthocyanin extracts showed a significant increase in the levels of fumarate, dimethylglycine, and 2-oxoglutarate and decreased levels of acetoin, alanine, 3-methyl-2-oxovalerate, and lactate compared to the M group (Figure 2C–F, Table S2). AAPP decreased the benzoate level (Figure 2E–F).

As the diabetic state progressed, the M group showed a significant difference in the levels of a larger number of metabolites compared to the lean Zucker rats at week 4 than that at week 1. Decreased levels of citrate, creatinine, trigonelline, hippurate, fumarate, *N*-methylnicotinamide, dimethylamine, and phenylalanine and higher levels of glucose, mannose, arabinose, the unknown metabolite, ethanol, formate, and trimethylamine were observed in ZDF rats compared to the lean Zucker rats (the ND and Con groups) (Figure 3A–B). NAAB increased the levels of 2-oxoglutarate, *N*-methylnicotinamide, and trigonelline, whereas L-AAPP increased the content of acetate. High doses of both anthocyanin extracts (H-AAPP and H-NAAB) increased the concentration (i.e., bin integral associated with a metabolite) of citrate, hippurate, and phenylalanine in the urine. In addition, H-AAPP also decreased the level of glucose, arabinose, lactate, ethanol, and formate (Figure 3C–F).

The anthocyanin extracts showed less effect on the urinary metabolites at week 8 compared to the extent of metabolic change at week 4. Most of the altered metabolites in the M group compared to the lean Zucker rats at week 8 were similar to those at week 4, apart from the levels of fumarate and trimethylamine, which were no longer significantly different between the lean Zucker rats and the M group at week 8 (Figure S2A–B). The nonacylated anthocyanin extracts (H-NAAB and L-NAAB) increased the content of 2-oxoglutarate, whereas a high-dose of acylated anthocyanin extract (H-AAPP) decreased the level of formate (Figure S2C–F).

### Metabolic Pathway Analysis

**Figure 4:**
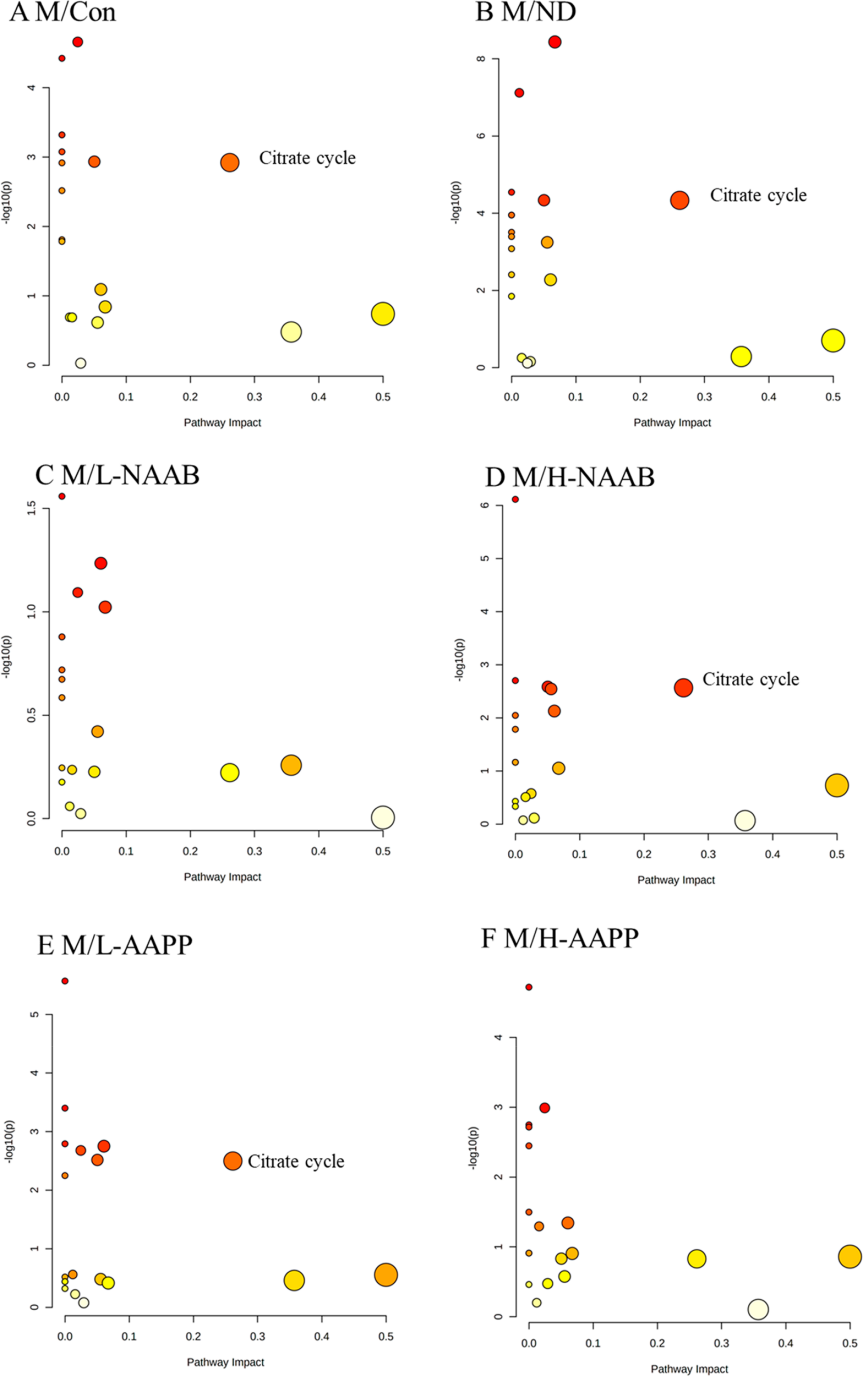
Metabolic pathway analysis generated with the MetaboAnalyst software package based on urine metabolites at week 1, showing altered pathways in M/Con (A), M/ND (B), M/L-NAAB (C), M/H-NAAB (D), M/L-AAPP (E), and M/H-AAPP (F) comparisons. The *p*-values in the *Y*-axis are generated from the pathway enrichment analysis, and the *X*-axis presents the pathway impact values from pathway topology analysis. The node color indicates the *p*-value from the pathway enrichment analysis (more reddish color indicates more significant changes in the pathway), whereas the node size reflects the pathway impact score. Pathways with small *p*-values and large pathway impact scores are considered as highly influential.

**Figure 5:**
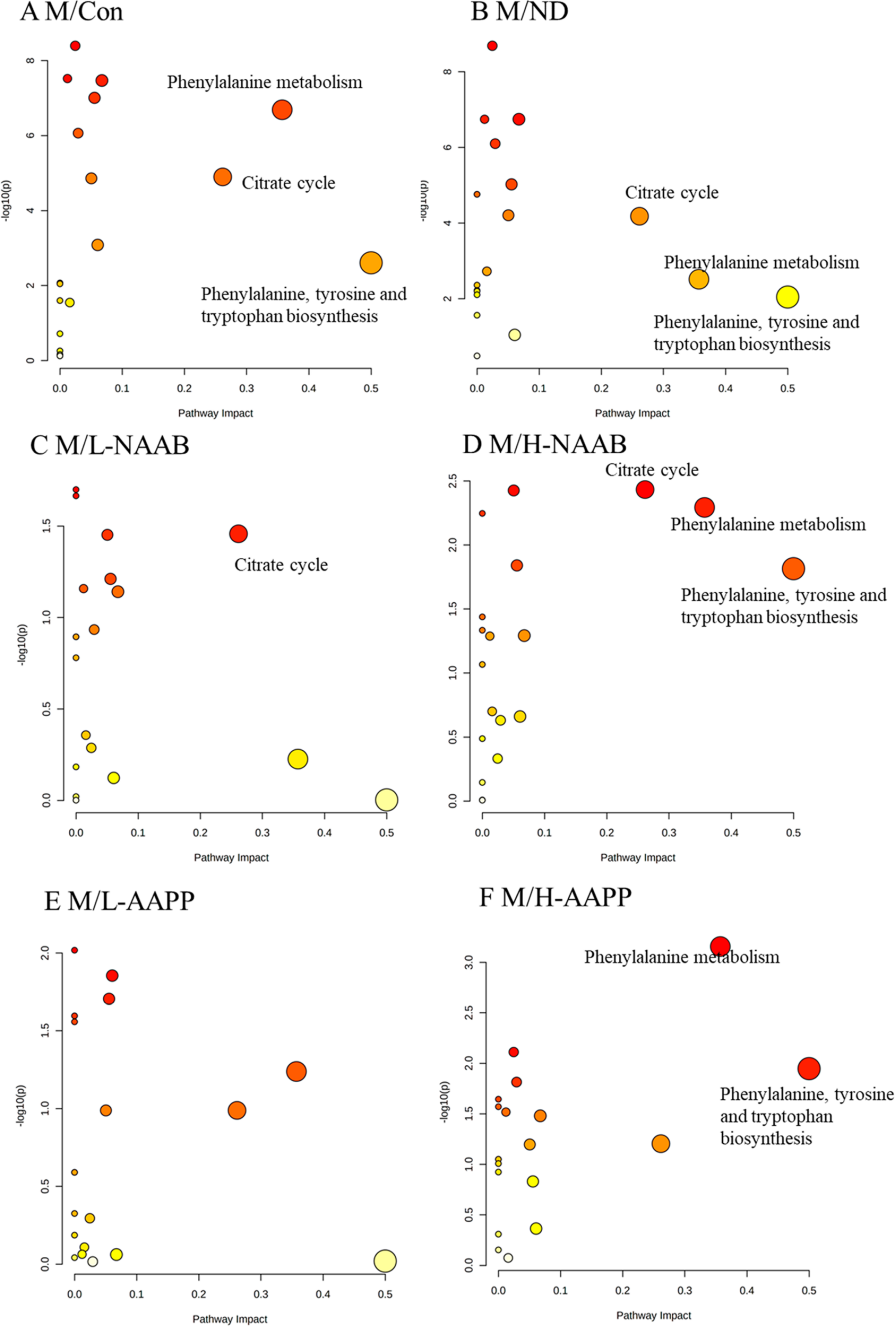
Metabolic pathway analysis generated with the MetaboAnalyst software package based on urine metabolites at week 4, showing altered pathways in M/Con (A), M/ND (B), M/L-NAAB (C), M/H-NAAB (D), M/L-AAPP (E), and M/H-AAPP (F) comparisons. The *p*-values in the *Y*-axis are generated from the pathway enrichment analysis, and the *X*-axis presents the pathway impact values from pathway topology analysis. The node color indicates the *p*-value from the pathway enrichment analysis (more reddish color indicates more significant changes in the pathway), whereas the node size reflects the pathway impact score. Pathways with small *p*-values and large pathway impact scores are considered highly influential.

The citrate cycle pathway was affected in the M groups compared to the ND and Con groups at week 1 as shown in Figure 4. H-NAAB and L-AAPP affected the citrate cycle pathway by mainly increasing the citrate and 2-oxoglutarate levels (Figure 4). At week 4, phenylalanine metabolism, citrate cycle, and phenylalanine, tyrosine, and tryptophan biosynthesis were changed in the diabetic M group in comparison to the lean Zucker rats. H-NAAB affected all those pathways, and H-AAPP affected phenylalanine metabolism and phenylalanine, tyrosine and tryptophan biosynthesis, which were mainly contributed by the increased phenylalanine level (Figure 5). At week 8, the same changed metabolic pathways were observed in the diabetic M group at week 4 in comparison to the lean Zucker rats; however, no pathways were affected by anthocyanin extracts (Figure S3).

### Dynamic Changes in Urinary Metabolomic Profile in ZDF Rats and Effects of Anthocyanin Extracts

**Figure 6:**
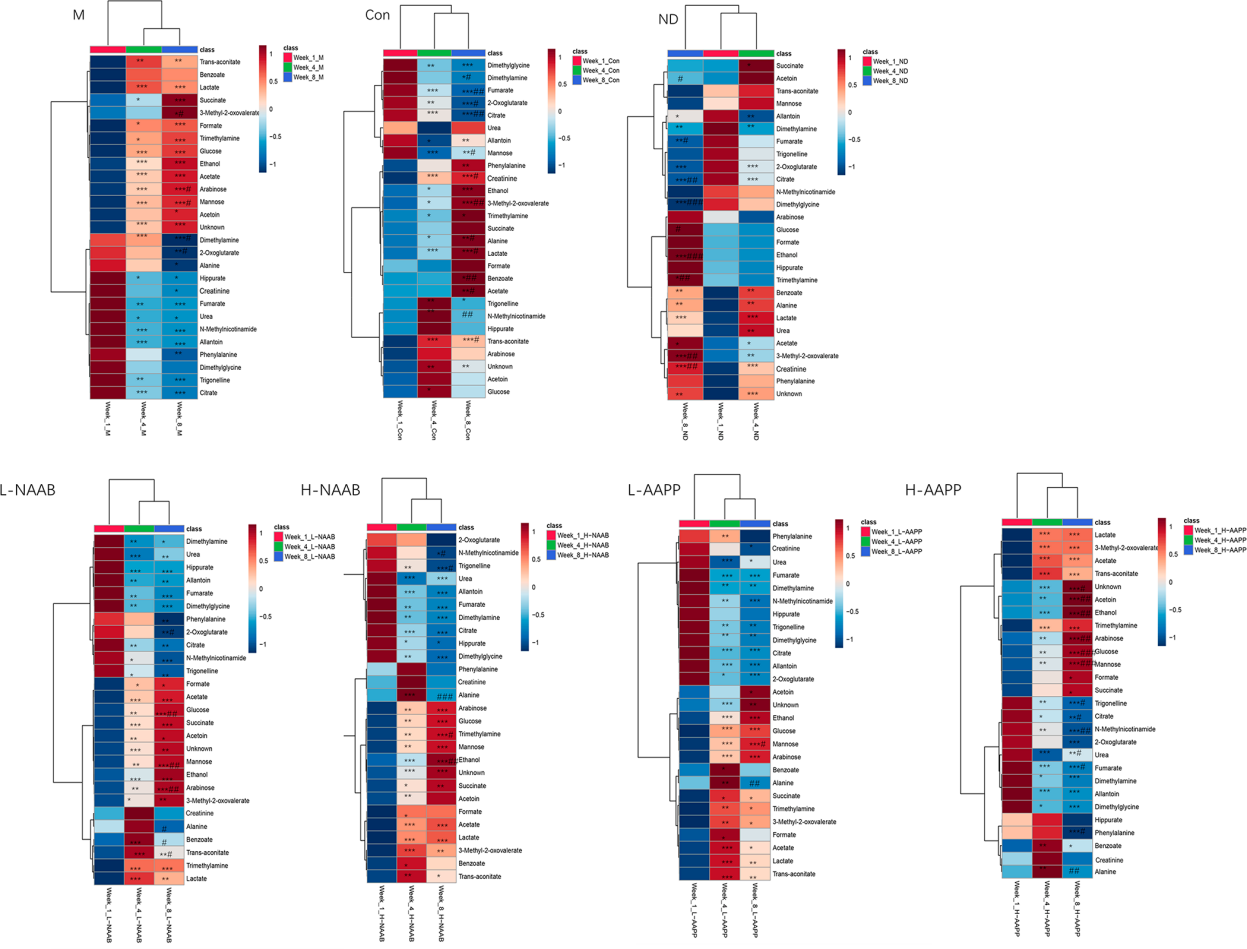
Alterations of urinary metabolites during the intervention time from week 1 to week 8 in each group. *p < 0.05, **p < 0.01, and ***p < 0.001 as compared with the week 1 time point, #*p* < 0.05, ##*p* < 0.01, and ###*p* < 0.001 as compared with the week 4 time point. Unknown indicates the unknown metabolite in the 1.13 to 1.14 ppm region of ^1^H NMR spectra.

Dynamic changes of urinary metabolomic profile in ZDF rats compared to lean Zucker rats from 4-week age to 12-week age were shown for the first time in this study, which help us to understand the effect of the defect in the *leptin* receptor gene and the modulatory effect of anthocyanins on the development of T2D. The alterations of metabolites are presented in the heat maps (Figure 6) and line charts (week 1, week 4, and week 8) (Figure S4).

We divided the metabolites into two categories in each group: metabolites with an increasing trend or decreasing trend during the development of diabetes. The metabolites with an increasing trend were increased significantly from week 1 to week 4 or/and 8 and *vice versa* for the metabolites with decreasing trend.

The increasing trend of acetate, trimethylamine, lactate, 3-methyl-2-oxovalerate, ethanol, and the unknown metabolite as well as the decreasing trend of citrate, 2-oxoglutarate, dimethylamine, dimethylglycine, and fumarate were found in all groups (Figure 6 and Figure S4), regardless of the genetic background and diet fed, which might have been associated with the growth and development of the rats. Among these metabolites, the levels of the unknown metabolite, trimethylamine, 3-methyl-2-oxovalerate, and ethanol were elevated in the ZDF rats compared to the lean Zucker rats at weeks 4 and 8. An increasing trend of creatinine and alanine was only found in the ND and Con groups, whereas the *leptin* receptor gene defect induced a large increase in acetoin, glucose, mannose, arabinose, and formate and a decrease in trigonelline and *N*-methylnicotinamide in the ZDF rats (Figure S4). High-doses of anthocyanin extracts (H-NAAB and H-AAPP) promoted a slightly increasing trend of benzoate and mitigated the increased trend of phenylalanine and indoxyl sulfate in diabetes, which occurred in lean Zucker rats. Another interesting finding was that the decreasing trend of hippurate, which occurred in the M group, was depressed by acylated anthocyanin extracts (AAPP) (FigureS4)

### Correlation Network of Plasma and Urine Metabolites

**Figure 7:**
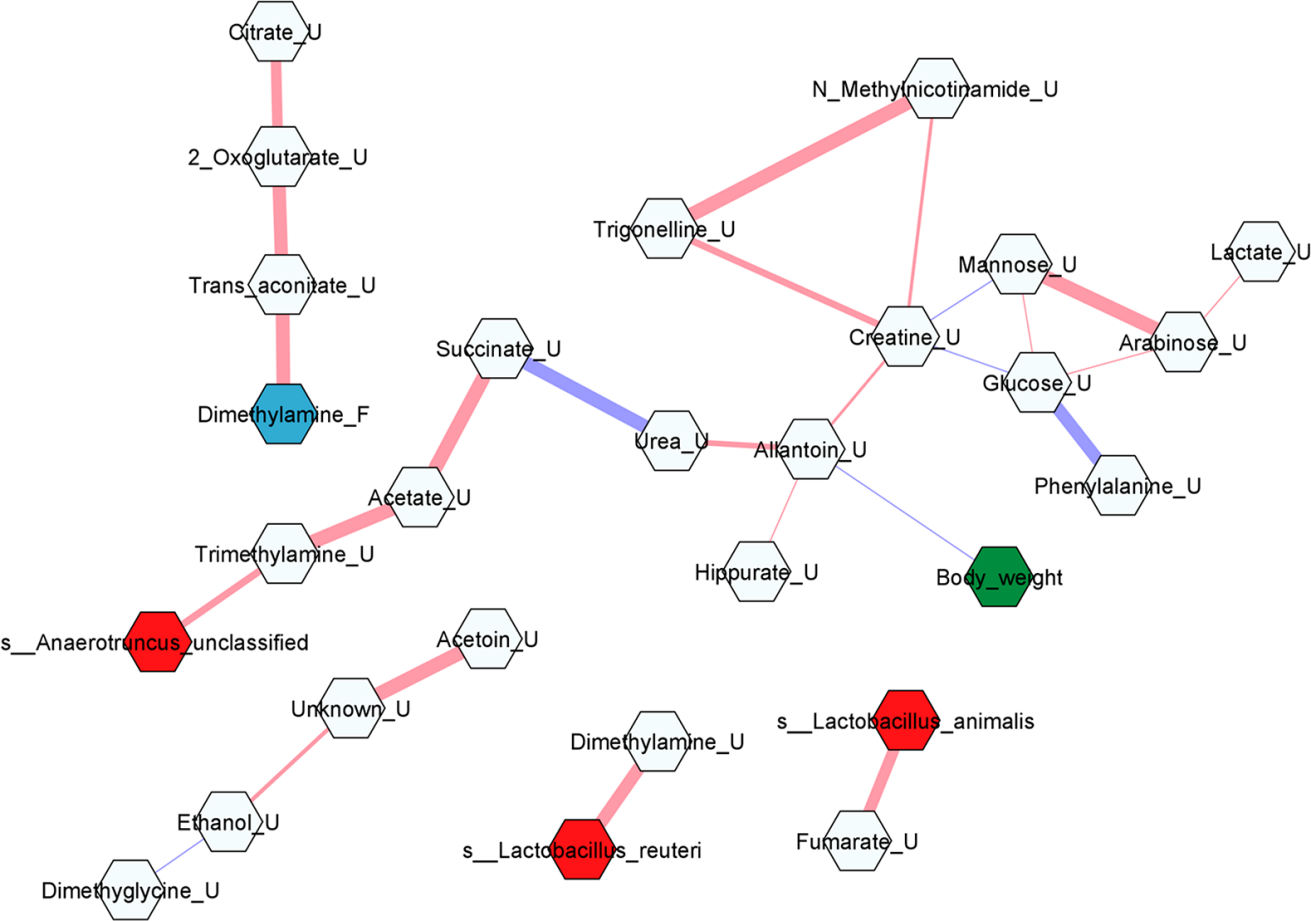
Correlation network constructed from urinary metabolites at week 8 in this study, plasma, hepatic, and fecal metabolites, and fecal microbiota as well as clinical traits reported from our previous study. The relevant network was shown with debiased sparse partial analysis (*r* > 0.4 or *r* < −0.4 and *p* < 0.05). The light blue hexagons indicate urinary metabolites. The cyan hexagon indicates fecal metabolites. The green hexagon indicates the clinical traits. The red hexagons indicate the gut microbiota. The color gradient of edges between nodes indicates the positive (red) and negative (purple) correlation. Edge thickness indicates the range of *p*-value. The thickest edge indicates the smallest *p*-value.

We have reported metabolites from plasma, liver, and feces and fecal microbiota as well as clinical traits in our previous studies.^6−8^ To explore the potential correlations of urinary metabolites to those variables and correlations between urinary metabolites, a correlation network was constructed (Figure 7).

Among the urinary metabolites, 2-oxoglutarate was positively correlated with citrate and *trans*-aconitate; trigonelline was positively correlated with creatinine and *N*-methylnicotinamide; and the glucose level showed a positive correlation with arabinose and mannose and a negative correlation with phenylalanine and creatinine. Urinary allantoin was negatively correlated with the body weight of the rats. Urinary *trans*-aconitate was positively correlated with fecal dimethylamine. Positive correlations were found between urinary metabolites and fecal microbiota: trimethylamine with *Anaerotruncus* sp.; urinary dimethylamine with *Lactobacillus reuteri*; urinary fumarate with *Lactobacillus animalis*. However, no correlation was found between urinary metabolites and plasma or hepatic metabolites.

## Discussion

We previously have studied metabolic profiles on plasma, liver, and gut to study different types of anthocyanins on T2D at the end of the intervention (week 8).^6−8^ Noninvasive measurement of urinary metabolites enables us to identify the dynamic changes in metabolites at different time points during the intervention period (weeks 1, 4, and 8). In the current study, ^1^H NMR metabolomics was used to investigate the dynamic alterations of the urinary metabolites in ZDF rats and the effects of nonacylated anthocyanins and acylated anthocyanins extracts.

The disturbed urinary metabolic profile of diabetic ZDF rats was observed at week 1 (5-week of age), and this disturbance was aggravated at weeks 4 and 8 as reflected by the increased number of metabolites showing a significant difference between the lean Zucker rats and the ZDF rats. Both types of anthocyanin extracts showed modulatory or reversing effects on the abnormal profile of urine metabolites in ZDF rats. This modulatory effect was observed in all anthocyanin-treated groups at weeks 1 and 4, as indicated by the larger number of metabolites significantly altered. Fewer metabolites were altered by anthocyanin extracts at week 8 compared to the previous two time points, which might indicate a metabolic change induced by the progression of T2D counteracted the beneficial effect of anthocyanins.

2-Oxoglutarate and fumarate as the TCA cycle intermediates were decreased in *leptin* gene receptor defect-induced diabetes (M vs Con) at both weeks 1 and 4, and this difference disappeared later at week 8. For other intermediates of the TCA cycle, succinate was decreased at week 1, and citrate and *trans*-aconitate appeared to be lower at weeks 4 and 8 in the diabetic M group than in lean Zucker rats (the ND and Con groups). A similar decreasing trend of TCA cycle flux (succinate, 2-oxoglutarate, and citrate) has also been observed in ZDF rats aged 8 weeks in a previous study.^29^ These results indicated a low rate of energy turnover through the TCA cycle at the early stage of diabetes in ZDF rats (weeks 1 and 4 corresponding to 5-week of age and 8-week of age, respectively), and these metabolites involved in the TCA cycle were further disturbed later (at the week 8 time point corresponding to 12-weeks of age). Our previous studies^6−8^ have shown all identified metabolites involved in the TCA cycle, such as plasma citrate, hepatic malate, and succinate as well as fecal succinate, were increased in ZDF rats at week 8. However, the alterations of TCA intermediates differ depending on the type of diabetic model,^30^ types of samples (serum, urine, or tissue),^31,32^ and fasting state as well as the stages of type 2 diabetes.^31^ For example, a reduced muscular TCA cycle flux in type 2 diabetic patients, reflecting mitochondrial dysfunction, has been observed.^32^ In contrast, *db/db* mice have shown higher levels of serum malate, citrate, aconitate, and succinate which are involved in the TCA cycle at 6-weeks of age than healthy mice, and these metabolites declined rapidly at 8-weeks of age;^31^ however, urinary levels of malate and succinate have been observed to be increased at 10-weeks of age and then decreased from 12-weeks of age to 16-weeks of age.^31^ Both types of anthocyanins increased TCA cycle intermediates levels (*trans*-aconitate, 2-oxoglutarate, citrate, and fumarate), which were lower in the M group at weeks 1 and 4 than the Con group. Thus, a modulatory effect of anthocyanins on metabolites involved in the TCA cycle was observed, suggesting an improved TCA cycle in ZDF rats. Those changes in energy metabolites might be due to the altered activity of AMP-activated protein kinase (AMPK) which is an important energy-sensing pathway to triggering glucose utilization and other energy metabolisms, and AMPK has been widely reported to be activated by different types of anthocyanins.^33^ Metabolic analysis shows an altered citrate cycle pathway was already observed as early as at week 1 and continued to be affected at weeks 4 and 8 in the M group compared to the ND and Con groups. H-NAAB and L-AAPP affected the citrate cycle pathway at week 1; NAAB affected the citrate cycle pathway at week 4. Among the detected TCA cycle intermediates (fumarate, 2-citrate, oxoglutarate, succinate, and *trans*-aconitate), only 2-oxoglutarate was positively correlated with citrate and *trans*-aconitate, indicating fumarate and succinate to be likely affected by other metabolisms, such as gluconeogenesis. 2-Oxoglutarate was positively correlated with citrate and *trans*-aconitate, indicating the consistent change of these metabolites as TCA cycle intermediates in diabetes.

Increased urinary content of alanine has been associated with enhanced hepatic glucose production and initial tubular damage of the kidney in diabetes.^34,35^ In the current study, the level of alanine was higher in the M group than in the lean Zucker rats at week 1, and both anthocyanin extracts reversed this increase. However, the level of alanine was lower in the M group at week 8 than the levels in the lean Zucker rats, indicating a more complicated role of alanine in diabetes, which deserves further study. In our previous studies on hepatic metabolomics and transcriptomics, the levels of dimethylglycine and *Bhmt* gene encoding betaine-homocysteine *S*-methyl-transferase responsible for the synthesis of dimethylglycine were higher in the M group than the Con group and both the anthocyanin extracts significantly decreased the level of hepatic dimethylglycine and expression of *Bhmt* gene at week 8.^7^ In this study, however, lower urinary level of dimethylglycine in ZDF rats at all time points was detected compared to the level in the lean Zucker rats, which was reversed by all anthocyanin extracts at weeks 1 and 8 (Table S4). Dimethylglycine participates in the cellular methionine and homocysteine cycle mechanism.^10^ Moderate intake of dietary anthocyanin has been reported to decrease homocysteine, which is an independent biomarker of inflammation in cardiovascular thrombotic disease.^36^ Although homocysteine was not identified by ^1^H NMR, changed dimethylglycine by anthocyanins might affect the inflammatory status by influencing homocysteine synthesis.

The contents of trigonelline, *N*-methylnicotinamide, hippurate, dimethylamine, and phenylalanine were lower in the M group compared to the levels in the lean Zucker rats at weeks 4 and 8. *N*-Methylnicotinamide and trigonelline are the methylated metabolites of niacin and nicotinamide;^37^ trigonelline also showed a positive correlation with *N*-methylnicotinamide, indicating methylation of niacin and nicotinamide is co-occurrent. Many studies have shown the positive effects of trigonelline on T2D. Diabetic Goto-kakizaki rats have shown decreased insulin resistance and lowered triglyceride levels after administration of trigonelline, which might be due to the modulatory effect of trigonelline increasing the activity of hepatic glucokinase and carnitine palmitoyl transferase as well as lowering hepatic fatty acid synthase.^38^ In addition, trigonelline intake has led to a positive effect shown as a reduction of early glucose and insulin responses revealed by oral glucose tolerance test in overweight men.^39^ The antidiabetic mechanisms of trigonelline have been summarized as improving β cell regeneration and insulin secretion as well as regulating key enzymes related to reactive oxygen species and glucose metabolism.^40^ In this study, both types of anthocyanins (particularly, L-NAAB, H-NAAB, and H-AAPP) significantly increased trigonelline levels at week 4, which is suggestive of the protection activity of these anthocyanin extracts in diabetes by modulating key enzymes related to energy metabolism.^41^ The concentrations of urinary hippurate, dimethylamine, and trimethylamine are associated with the activities and composition of microbes in the gut,^42^ and changes in these metabolites indicate altered gut microbiota. A lower level of hippurate was seen in the diabetic M group compared to lean Zucker rats at weeks 4 and 8; a similar change was also verified in T2D patients^30^ and ZDF rats,^43^ which might be related to the “obese microbiome”.^44^ Our previous study in fecal microbiota has shown that several diabetes- and obesity-related species, *Clostridium hathewayi*,^45^*Lachnospiraceae* spp.,^45^ and *Akkermansia muciniphila*,^46^ have been altered in the M group compared to the Con and ND groups. Furthermore, reduced urinary hippurate level has been reported in certain renal disorders, which might be associated with diabetic renal dysfunction. For example, reduced hippurate is an early biomarker of nephrotoxicity and renal tubular malfunction in rats^47^ and in humans.^48^ Both types of anthocyanins at high dose increased hippurate at week 4, indicating a protective effect on T2D and/or diabetic renal function. Our previous postprandial clinical study has shown that hippurate was the most abundant urinary metabolite derived from dietary anthocyanins due to the fact that it is formed as a detoxification product of aromatic compounds,^49^ which also partly contributed to the increase of hippurate level. In addition, the availability of hepatic acetyl-CoA, a rate-limiting factor of synthesizing hippurate, might be increased by anthocyanins extracts.^50^ Trimethylamine and dimethylamine are metabolites involved in choline metabolism by gut microbiota. Dietary choline is first converted to trimethylamine, which can be further degraded to dimethylamine in the gut.^51^ In this study, the level of trimethylamine was higher in the M group than in lean Zucker rats at week 4, and this increase disappeared at week 8. The urinary level of trimethylamine was shown to be positively correlated to the abundance of species *Anaerotruncus* sp., which has also been reported in patients with atherosclerotic and cardioembolic strokes, suggesting *Anaerotruncus* sp. might have a significant role in trimethylamine production which promotes the development of cardiovascular diseases.^52^ Another human study with 24 individuals susceptible to developing metabolic syndrome has shown a positive relationship between *Anaerotruncus* sp. and trimethylamine *N*-oxide which can be formed from trimethylamine in the liver.^53^ The level of dimethylamine was lower in the M group at weeks 4 and 8. We only observed that H-NAAB and H-AAPP increased dimethylamine at week 1, indicating an immediate effect of anthocyanin extracts on choline metabolism after a short period of intervention. In the current study, the urinary level of dimethylamine at week 8 was found to be positively correlated to *Lactobacillus reuteri*. *Lactobacillus reuteri* as a probiotic has a beneficial effect on glucose metabolism in T2D, possibly by regulating glucose transporter 5 and Na^+^-coupled glucose transporter in animal models.^54,55^ Lowered urinary dimethylamine has also been verified in T2D patients.^56^ However, dimethylamine was only increased by a high dose of anthocyanin extracts at week 1, indicating a possible improvement of early stage T2D by the intervention of anthocyanins via modulating gut microbiota and glucose transporters. The phenylalanine level was decreased in the M group at weeks 4 and 8 and negatively correlated with glucose level. Decreased urinary phenylalanine level has been verified in subjects with T2D^57,58^ and considered as an important biomarker for diabetes progression.^58^ H-AAPP increased the phenylalanine level at week 4. Metabolic analysis revealed that phenylalanine, tyrosine, and tryptophan biosynthesis and phenylalanine metabolism changed at week 4 in the M group compared to the Con and ND groups and sustained until the end of the experiment at week 8. Only high doses of NAAB and AAPP affected these metabolisms at week 4. The decay of phenylalanine metabolism and phenylalanine, tyrosine, and tryptophan biosynthesis the in T2D model could be attributed to the increased amino acid utilization for gluconeogenesis as a result of the impaired β-cell capacity to produce insulin and/or the compromised insulin signaling.^58^ H-AAPP and H-NAAB might improve insulin signaling by regulating phenylalanine, tyrosine, and tryptophan biosynthesis and phenylalanine metabolism at week 4.

Formate, glucose, arabinose, mannose, and ethanol were higher in the M group at weeks 4 and 8 in ZDF rats compared to the lean Zucker rats, and only a high dose of acylated anthocyanins (H-AAPP) reversed the increase in formate, glucose, arabinose, and ethanol in ZDF rats at week 4. A higher level of formate has been reported to inhibit terminal electron acceptors of the electron transport chain and disrupt energy production.^59^ Formate could be produced from methanol in the liver by mitochondrial alcohol dehydrogenase, which induces the production of free radicals; alternatively, formate can also be produced by intestinal bacteria.^59^ Mulberry anthocyanin extract has been reported to regulate mitochondrial function, such as increasing mitochondrial size, energy production, and mitochondrial DNA content in high-fat diet-induced obese rats.^60^ H-AAPP decreased formate, suggesting a possible improvement in energy production and mitochondrial function at week 4; this decrease caused by H-AAPP disappeared at week 8, which might indicate that the progression of T2D neutralized the beneficial effect of anthocyanins on the formate production. A high level of urinary glucose in ZDF rats indicates a high level of plasma glucose and/or renal dysfunction. Our previous studies have shown acylated anthocyanins from purple potato have more potential to decrease plasma and hepatic glucose levels,^6,7^ and in this study, only a high dose of acylated anthocyanin extracts (AAPP) decreased urinary glucose. Urinary lactate level was higher in the diabetic group than in the lean Zucker rats. Oxidative stress commonly in type 2 diabetes could stimulate lactate dehydrogenase and increase lactate production.^61^ Anthocyanins have been reported to activate the Nrf2/Keap1 pathway, which initiates the transcription of downstream genes coding antioxidant enzymes to resist oxidative stress.^62^ Due to anthocyanins having strong antioxidative activities and their ability to activate antioxidant enzymes,^62,63^ both types of anthocyanins decreased the lactate level at week 1 and only a high dose of acylated anthocyanin extracts (H-AAPP) decreased the lactate level as diabetes progressed to week 4, possibly through activating the Nrf2/Keap1 pathway. Correlation network analysis revealed the glucose level was positively correlated with levels of arabinose and mannose, indicating the consistent change of these monosaccharides, metabolisms in diabetes. These results indicate that a high dose of potato anthocyanin extract has more potential to improve energy production in mitochondria, glucose homeostasis, and oxidative stress at week 4.

Decreased urinary levels of allantoin in ZDF rats compared to lean Zucker rats were consistently observed from week 1 to week 8, which verified previous similar findings and was associated with altered renal tubular function in diabetes.^30,64^ A higher level of urea nitrogen in blood has also been detected in the ZDF rats in our previous study, also suggesting an altered renal function.^64^ Since the urinary allantoin level could accurately reflect the glomerular filtration rate (GFR),^30,65^ decreased allantoin in ZDF rats might be associated with diabetic nephropathy, possibly indicating low GFR. Another interesting finding was that the level of urinary allantoin was negatively correlated with body weight; this positive correlation might be due to the fact that allantoin could activate the imidazoline I1 receptor, which can regulate appetite and decrease energy intake and body weight.^66^ However, in this study anthocyanin extracts did not affect urinary allantoin levels.

In addition to the acylation of the anthocyanins in two extracts, other phenolic compounds, such as the chlorogenic acid in the purple potato anthocyanin extract, could have also affected the urinary metabolites.^59^

In summary, disturbance in the urinary metabolic profile of T2D in ZDF rats occurred already at week 1, and this disturbance aggravated at week 4 and relatively stabilized when the intervention time proceeded from week 4 to week 8. Both acylated and nonacylated anthocyanin extracts showed modulatory effects on urine metabolites; however, this modulatory effect weakened gradually as diabetes progressed. Both types of anthocyanins modulated the levels of 2-oxoglutarate, fumarate, alanine, acetoin, and dimethylglycine at week 1, and a high dose of both anthocyanin extracts increased citrate, trigonelline, hippurate, and phenylalanine at week 4, suggesting a possible modulating effect on the TCA cycle, choline and betaine metabolism, gut microbiota, renal function, and antioxidative capacity. In addition, only a high dose of acylated anthocyanins from potatoes decreased glucose, arabinose, lactate, ethanol, and formate in the urine, which were increased in the ZDF rats at weeks 1 and 4, suggesting a more potential improvement in energy production in mitochondria, glucose homeostasis, and oxidative stress compared to nonacylated anthocyanins.

## Supporting Information Available

^1^H chemical shift assignments of the metabolites, fold change of the urine metabolites, ^1^H NMR spectrum of urine sample, volcano plots showing the significantly different metabolites from urine at week 8, metabolic pathway analysis generated with the MetaboAnalyst software package based on urine metabolites at week 8, and line charts showing the metabolite levels at different time points (PDF)

## Abbreviations Used

AAPP: acylated anthocyanins extract from purple potatoes
NAAB: nonacylated anthocyanins extract from bilberries
PLS-DA: partial least-squares discriminant analysis
ROC: receiver operating characteristic
T2D: type 2 diabetes
TCA cycle: tricarboxylic acid cycle
ZDF: Zucker diabetic fatty

## References

[ref1] Cho N. H.; Shaw J. E.; Karuranga S.; Huang Y.; da Rocha Fernandes J. D.; Ohlrogge A. W.; Malanda B. IDF Diabetes Atlas: Global Estimates of Diabetes Prevalence for 2017 and Projections for 2045. Diabetes Res. Clin Pract 2018, 138, 271–281. 10.1016/j.diabres.2018.02.023. 29496507

[ref2] Koh E. S.; Lim J. H.; Kim M. Y.; Chung S.; Shin S. J.; Choi B. S.; Kim H. W.; Hwang S. Y.; Kim S. W.; Park C. W.; Chang Y. S. Anthocyanin-Rich Seoritae Extract Ameliorates Renal Lipotoxicity via Activation of AMP-Activated Protein Kinase in Diabetic Mice. J. Transl Med. 2015, 13 (1), 1–12. 10.1186/s12967-015-0563-4. 26116070 PMC4482313

[ref3] Xu J.; Su X.; Lim S.; Griffin J.; Carey E.; Katz B.; Tomich J.; Smith J. S.; Wang W. Characterisation and Stability of Anthocyanins in Purple-Fleshed Sweet Potato P40. Food Chem. 2015, 186, 90–96. 10.1016/j.foodchem.2014.08.123. 25976796

[ref4] Moriya C.; Hosoya T.; Agawa S.; Sugiyama Y.; Shin-ya K.; Terahara N.; Kumazawa S. New Acylated Anthocyanins from Purple Yam and Their Antioxidant Activity. Biosci Biotechnol Biochem 2015, 79, 1484–1492. 10.1080/09168451.2015.1027652. 25848974

[ref5] Nizamutdinova I. T.; Jin Y. C.; Chung J.; Shin S. C.; Lee S. J.; Seo H. G.; Lee J. H.; Chang K. C.; Kim H. J. The Anti-Diabetic Effect of Anthocyanins in Streptozotocin-Induced Diabetic Rats through Glucose Transporter 4 Regulation and Prevention of Insulin Resistance and Pancreatic Apoptosis. Mol. Nutr Food Res. 2009, 53 (11), 1419–1429. 10.1002/mnfr.200800526. 19785000

[ref6] Chen K.; Wei X.; Zhang J.; Pariyani R.; Jokioja J.; Kortesniemi M.; Linderborg K. M.; Heinonen J.; Sainio T.; Zhang Y.; Yang B. Effects of Anthocyanin Extracts from Bilberry (Vaccinium Myrtillus L.) and Purple Potato (Solanum Tuberosum L. Var. ‘Synkeä Sakari’) on the Plasma Metabolomic Profile of Zucker Diabetic Fatty Rats. J. Agric. Food Chem. 2020, 68 (35), 9436–9450. 10.1021/acs.jafc.0c04125. 32786839 PMC7586333

[ref7] Chen K.; Wei X.; Pariyani R.; Kortesniemi M.; Zhang Y.; Yang B. 1H NMR Metabolomics and Full-Length RNA-Seq Reveal Effects of Acylated and Nonacylated Anthocyanins on Hepatic Metabolites and Gene Expression in Zucker Diabetic Fatty Rats. J. Agric. Food Chem. 2021, 69, 4423–4437. 10.1021/acs.jafc.1c00130. 33835816 PMC8154569

[ref8] Chen K.; Wei X.; Kortesniemi M.; Pariyani R.; Zhang Y.; Yang B. Effects of Acylated and Nonacylated Anthocyanins Extracts on Gut Metabolites and Microbiota in Diabetic Zucker Rats : A Metabolomic and Metagenomic Study. Food Research International 2022, 153, 110978 10.1016/j.foodres.2022.110978. 35227465

[ref9] Bouatra S.; Aziat F.; Mandal R.; Guo A. C.; Wilson M. R.; Knox C.; Bjorndahl T. C.; Krishnamurthy R.; Saleem F.; Liu P.; Dame Z. T.; Poelzer J.; Huynh J.; Yallou F. S.; Psychogios N.; Dong E.; Bogumil R.; Roehring C.; Wishart D. S. The Human Urine Metabolome. PLoS One 2013, 8 ( (9), ), e73076 10.1371/journal.pone.0073076. 24023812 PMC3762851

[ref10] Hyeon J. S.; Jung Y.; Lee G.; Ha H.; Hwang G. S. Urinary Metabolomic Profiling in Streptozotocin-Induced Diabetic Mice after Treatment with Losartan. Int. J. Mol. Sci. 2020, 21 (23), 8969 10.3390/ijms21238969. 33255934 PMC7730544

[ref11] Williams R. E.; Lenz E. M.; Rantalainen M.; Wilson I. D. The Comparative Metabonomics of Age-Related Changes in the Urinary Composition of Male Wistar-Derived and Zucker (Fa/Fa) Obese Rats. Mol. Biosyst 2006, 2 (3–4), 193–202. 10.1039/b517195d. 16880937

[ref12] Liu H.; Tayyari F.; Khoo C.; Gu L. A 1H NMR-Based Approach to Investigate Metabolomic Differences in the Plasma and Urine of Young Women after Cranberry Juice or Apple Juice Consumption. J. Funct Foods 2015, 14, 76–86. 10.1016/j.jff.2015.01.018.

[ref13] Gomes A.; Godinho-Pereira J.; Oudot C.; Sequeira C. O.; Macià A.; Carvalho F.; Motilva M. J.; Pereira S. A.; Matzapetakis M.; Brenner C.; Santos C. N. Berry Fruits Modulate Kidney Dysfunction and Urine Metabolome in Dahl Salt-Sensitive Rats. Free Radic Biol. Med. 2020, 154 (April), 119–131. 10.1016/j.freeradbiomed.2020.05.002. 32437928

[ref14] Liu H.; Tayyari F.; Edison A. S.; Su Z.; Gu L. NMR-Based Metabolomics Reveals Urinary Metabolome Modifications in Female Sprague-Dawley Rats by Cranberry Procyanidins. Journal of Nutritional Biochemistry 2016, 34, 136–145. 10.1016/j.jnutbio.2016.05.007. 27309592 PMC8962502

[ref15] Emwas A. H.; Luchinat C.; Turano P.; Tenori L.; Roy R.; Salek R. M.; Ryan D.; Merzaban J. S.; Kaddurah-Daouk R.; Zeri A. C.; Nagana Gowda G. A.; Raftery D.; Wang Y.; Brennan L.; Wishart D. S. Standardizing the Experimental Conditions for Using Urine in NMR-Based Metabolomic Studies with a Particular Focus on Diagnostic Studies: A Review. Metabolomics 2015, 11 (4), 872–894. 10.1007/s11306-014-0746-7. 26109927 PMC4475544

[ref16] Terrettaz J.; Jeanrenaud B. In Vivo Hepatic and Peripheral Insulin Resistance in Genetically Obese (Fa/Fa) Rats. Endocrinology 1983, 112 (4), 1346–1351. 10.1210/endo-112-4-1346. 6339203

[ref17] Yan F.; Dai G.; Zheng X. Mulberry Anthocyanin Extract Ameliorates Insulin Resistance by Regulating PI3K/AKT Pathway in HepG2 Cells and Db/Db Mice. Journal of Nutritional Biochemistry 2016, 36, 68–80. 10.1016/j.jnutbio.2016.07.004. 27580020

[ref18] Wu T.; Qi X.; Liu Y.; Guo J.; Zhu R.; Chen W.; Zheng X.; Yu T. Dietary Supplementation with Purified Mulberry (Morus Australis Poir) Anthocyanins Suppresses Body Weight Gain in High-Fat Diet Fed C57BL/6 Mice. Food Chem. 2013, 141 (1), 482–487. 10.1016/j.foodchem.2013.03.046. 23768383

[ref19] Sarikaphuti A.; Nararatwanchai T.; Hashiguchi T.; Ito T.; Thaworanunta S.; Kikuchi K.; Oyama Y.; Maruyama I.; Tancharoen S. Preventive Effects of Morus Alba L. Anthocyanins on Diabetes in Zucker Diabetic Fatty Rats. Exp Ther Med. 2013, 6 (3), 689–695. 10.3892/etm.2013.1203. 24137248 PMC3786992

[ref20] Jokioja J. Anthocyanin-Rich Extract from Purple Potatoes Decreases Postprandial Glycemic Response and Affects Inflammation Markers in Healthy Men. Food Chem. 2020, 310, 125797 10.1016/j.foodchem.2019.125797. 31818516

[ref21] Lätti A. K.; Riihinen K. R.; Jaakola L. Phenolic Compounds in Berries and Flowers of a Natural Hybrid between Bilberry and Lingonberry (Vaccinium × Intermedium Ruthe). Phytochemistry 2011, 72 (8), 810–815. 10.1016/j.phytochem.2011.02.015. 21382629

[ref22] Gutiérrez-Quequezana L.; Vuorinen A. L.; Kallio H.; Yang B. Improved Analysis of Anthocyanins and Vitamin C in Blue-Purple Potato Cultivars. Food Chem. 2018, 242, 217–224. 10.1016/j.foodchem.2017.09.002. 29037681

[ref23] Dieterle F.; Ross A.; Senn H. Probabilistic Quotient Normalization as Robust Method to Account for Dilution of Complex Biological Mixtures. Application in 1 H NMR Metabonomics. Anal. Chem. 2006, 78, 4281–4290. 10.1021/ac051632c. 16808434

[ref24] Mora-Ortiz M.; Nuñez Ramos P.; Oregioni A.; Claus S. P. NMR Metabolomics Identifies over 60 Biomarkers Associated with Type II Diabetes Impairment in Db/Db Mice. Metabolomics 2019, 15 (6), 1–16. 10.1007/s11306-019-1548-8. PMC6556514 31179513

[ref25] Lamichhane S.; Yde C. C.; Schmedes M. S.; Jensen H. M.; Meier S.; Bertram H. C. Strategy for Nuclear-Magnetic-Resonance-Based Metabolomics of Human Feces. Anal. Chem. 2015, 87 (12), 5930–5937. 10.1021/acs.analchem.5b00977. 25985090

[ref26] Zhang L.; Dong M.; Xu G.; Tian Y.; Tang H.; Wang Y. Metabolomics Reveals That Dietary Ferulic Acid and Quercetin Modulate Metabolic Homeostasis in Rats. J. Agric. Food Chem. 2018, 66 (7), 1723–1731. 10.1021/acs.jafc.8b00054. 29359554

[ref27] Kortesniemi M.; Vuorinen A. L.; Sinkkonen J.; Yang B.; Rajala A.; Kallio H. NMR Metabolomics of Ripened and Developing Oilseed Rape (Brassica Napus) and Turnip Rape (Brassica Rapa). Food Chem. 2015, 172, 63–70. 10.1016/j.foodchem.2014.09.040. 25442524

[ref28] Chen K.; Wei X.; Zhang J.; Pariyani R.; Jokioja J.; Kortesniemi M.; Linderborg K.; Heinonen J.; Sainio T.; Zhang Y.; Yang B. Effects of Anthocyanin Extracts from Bilberry (Vaccinium Myrtillus L.) and Purple Potato (Solanum Tuberosum L. Var. ‘Synkeä Sakari’) on the Plasma Metabolomic Profile of Zucker Diabetic Fatty Rats. J. Agric. Food Chem. 2020, 68, 9436–9450. 10.1021/acs.jafc.0c04125. 32786839 PMC7586333

[ref29] Zhao L. C.; Zhang X. D.; Liao S. X.; Wang H. Y.; Lin D. H.; Gao H. C. A Metabonomic Comparison of Urinary Changes in Zucker and GK Rats. J. Biomed. Biotechnol. 2010, 2010, 431894 10.1155/2010/431894. 20981252 PMC2963802

[ref30] Salek R. M.; Maguire M. L.; Bentley E.; Rubtsov D. V.; Hough T.; Cheeseman M.; Nunez D.; Sweatman B. C.; Haselden J. N.; Cox R. D.; Connor S. C.; Griffin J. L. A Metabolomic Comparison of Urinary Changes in Type 2 Diabetes in Mouse, Rat, and Human. Physiol Genomics 2007, 29 (2), 99–108. 10.1152/physiolgenomics.00194.2006. 17190852

[ref31] Li M.; Wang X.; Aa J.; Qin W.; Zha W.; Ge Y.; Liu L.; Zheng T.; Cao B.; Shi J.; Zhao C.; Wang X.; Yu X.; Wang G.; Liu Z. GC/TOFMS Analysis of Metabolites in Serum and Urine Reveals Metabolic Perturbation of TCA Cycle in Db/Db Mice Involved in Diabetic Nephropathy. Am. J. Physiol. Renal Physiol. 2013, 304 (11), F1317–F1324. 10.1152/ajprenal.00536.2012. 23467425

[ref32] Schrauwen P.; Hesselink M. K. C. Reduced Tricarboxylic Acid Cycle Flux in Type 2 Diabetes Mellitus?. Diabetologia 2008, 51 (9), 1694–1697. 10.1007/s00125-008-1069-x. 18587560 PMC2516188

[ref33] Gowd V.; Jia Z.; Chen W. Anthocyanins as Promising Molecules and Dietary Bioactive Components against Diabetes e A Review of Recent Advances. Trends Food Sci. Technol. 2017, 68, 1–13. 10.1016/j.tifs.2017.07.015.

[ref34] Mutter S.; Valo E.; Aittomäki V.; Nybo K.; Raivonen L.; Thorn L. M.; Forsblom C.; Sandholm N.; Würtz P.; Groop P. H. Urinary Metabolite Profiling Identifies Biomarkers for Risk of Progression of Diabetic Nephropathy in 2,670 Individuals with Type 1 Diabetes. medRxiv 2020, 1–31. 10.1101/2020.10.21.20215921. PMC8660744 34686904

[ref35] Messana I.; Forni F.; Ferrari F.; Rossi C.; Giardina B.; Zuppi C. Proton Nuclear Magnetic Resonance Spectral Profiles of Urine in Type II Diabetic Patients. Clin Chem. 1998, 44 (7), 1529–1534. 10.1093/clinchem/44.7.1529. 9665433

[ref36] Bhatt N.; Waly M. I.; Ali A. Anti-Inflammatory Role of Anthocyanins in the Prevention of Hyperhomocysteinemia-Mediated Cardiometabolic Diseases. Nutritional Management and Metabolic Aspects of Hyperhomocysteinemia 2021, 33–49. 10.1007/978-3-030-57839-8_3.

[ref37] Abu Bakar Sajak A.; Mediani A.; Maulidiani; Mohd Dom N. S.; Machap C.; Hamid M.; Ismail A.; Khatib A.; Abas F. Effect of Ipomoea Aquatica Ethanolic Extract in Streptozotocin (STZ) Induced Diabetic Rats via 1H NMR-Based Metabolomics Approach. Phytomedicine 2017, 36, 201–209. 10.1016/j.phymed.2017.10.011. 29157816

[ref38] YOSHINARI O.; SATO H.; IGARASHI K. Anti-Diabetic Effects of Pumpkin and Its Components, Trigonelline and Nicotinic Acid, on Goto-Kakizaki Rats. Biosci Biotechnol Biochem 2009, 73 (5), 1033–1041. 10.1271/bbb.80805. 19420712

[ref39] Van Dijk A. E.; Olthof M. R.; Meeuse J. C.; Seebus E.; Heine R. J.; Van Dam R. M. Acute Effects of Decaffeinated Coffee and the Major Coffee Components Chlorogenic Acid and Trigonelline on Glucose Tolerance. Diabetes Care 2009, 32 (6), 1023–1025. 10.2337/dc09-0207. 19324944 PMC2681030

[ref40] Zhou J.; Chan L.; Zhou S. Trigonelline: A Plant Alkaloid with Therapeutic Potential for Diabetes and Central Nervous System Disease. Curr. Med. Chem. 2012, 19 (21), 3523–3531. 10.2174/092986712801323171. 22680628

[ref41] Lin M.; Xie Z.; Zhou Y.; Li Y.; Ren J.; Peng X. X.; Yao M.; Yang Z.; Liao Q. Dynamic Metabonomic and Microbiological Response of Rats to Lincomycin Exposure: An Integrated Microbiology and Metabonomics Analysis. RSC Adv. 2015, 5 (80), 65415–65426. 10.1039/C5RA10626E.

[ref42] Gooda Sahib Jambocus N.; Saari N.; Ismail A.; Khatib A.; Mahomoodally M. F.; Abdul Hamid A. An Investigation into the Antiobesity Effects of Morinda Citrifolia L. Leaf Extract in High Fat Diet Induced Obese Rats Using a 1H NMR Metabolomics Approach. J. Diabetes Res. 2016, 2016, 2391592 10.1155/2016/2391592. 26798649 PMC4698747

[ref43] Calvani R.; Miccheli A.; Capuani G.; Tomassini Miccheli A.; Puccetti C.; Delfini M.; Iaconelli A.; Nanni G.; Mingrone G. Gut Microbiome-Derived Metabolites Characterize a Peculiar Obese Urinary Metabotype. Int. J. Obes. 2010, 34 ( (6), ), 1095–1098. 10.1038/ijo.2010.44. 20212498

[ref44] Lees H. J.; Swann J. R.; Wilson I. D.; Nicholson J. K.; Holmes E. Hippurate: The Natural History of a Mammalian-Microbial Cometabolite. J. Proteome Res. 2013, 12 (4), 1527–1546. 10.1021/pr300900b. 23342949

[ref45] Wang J.; Qin J.; Li Y.; Cai Z.; Li S.; Zhu J.; Zhang F.; Liang S.; Zhang W.; Guan Y.; Shen D.; Peng Y.; Zhang D.; Jie Z.; Wu W.; Qin Y.; Xue W.; Li J.; Han L.; Lu D.; Wu P.; Dai Y.; Sun X.; Li Z.; Tang A.; Zhong S.; Li X.; Chen W.; Xu R.; Wang M.; Feng Q.; Gong M.; Yu J.; Zhang Y.; Zhang M.; Hansen T.; Sanchez G.; Raes J.; Falony G.; Okuda S.; Almeida M.; Lechatelier E.; Renault P.; Pons N.; Batto J. M.; Zhang Z.; Chen H.; Yang R.; Zheng W.; Li S.; Yang H.; Ehrlich S. D.; Nielsen R.; Pedersen O.; Kristiansen K.; Wang J. A Metagenome-Wide Association Study of Gut Microbiota in Type 2 Diabetes. Nature 2012, 490 (7418), 55–60. 10.1038/nature11450. 23023125

[ref46] Gurung M.; Li Z.; You H.; Rodrigues R.; Jump D. B.; Morgun A.; Shulzhenko N. Role of Gut Microbiota in Type 2 Diabetes Pathophysiology. EBioMedicine 2020, 51, e102590 10.1016/j.ebiom.2019.11.051. PMC6948163 31901868

[ref47] Bertram H. C.; Duus J. Ø.; Petersen B. O.; Hoppe C.; Larnkjær A.; Schack-nielsen L.; Mølgaard C.; Michaelsen K. F. Nuclear Magnetic Resonance - Based Metabonomics Reveals Strong Sex Effect on Plasma Metabolism in 17-Year - Old Scandinavians and Correlation to Retrospective Infant Plasma Parameters. Metabolism 2009, 58 (7), 1039–1045. 10.1016/j.metabol.2009.03.011. 19411084

[ref48] Bairaktari E.; Seferiadis K.; Liamis G.; Psychogios N.; Tsolas O.; Elisaf M. Rhabdomyolysis-Related Renal Tubular Damage Studied by Proton Nuclear Magnetic Resonance Spectroscopy of Urine. Clin. Chem. 2002, 48 (7), 1106–1109. 10.1093/clinchem/48.7.1106. 12089184

[ref49] Jokioja J.; Percival J.; Philo M.; Yang B.; Kroon P. A.; Linderborg K. M. Phenolic Metabolites in the Urine and Plasma of Healthy Men After Acute Intake of Purple Potato Extract Rich in Methoxysubstituted Monoacylated Anthocyanins. Mol. Nutr Food Res. 2021, 65 (9), 2000898 10.1002/mnfr.202000898. 33687145

[ref50] Lees H. J.; Swann J. R.; Wilson I. D.; Nicholson J. K.; Holmes E. Hippurate: The Natural History of a Mammalian-Microbial Cometabolite. J. Proteome Res. 2013, 12 (4), 1527–1546. 10.1021/pr300900b. 23342949

[ref51] Chhibber-Goel J.; Gaur A.; Singhal V.; Parakh N.; Bhargava B.; Sharma A. The Complex Metabolism of Trimethylamine in Humans: Endogenous and Exogenous Sources. Expert Rev. Mol. Med. 2016, 18, E8 10.1017/erm.2016.6. 27126549

[ref52] Xu D.-J.; Wang K.; Yuan L.-B.; Li H.-F.; Xu Y.-Y.; Wei L.-Y.; Chen L.; Jin K.-K. Dysbiosis of Gut Microbiota with Increased Trimethylamine N-Oxide Level in Patients with Large Artery Atherosclerotic and Cardioembolic Strokes. Research Square 2020, 10.21203/rs.3.rs-22813/v1.

[ref53] Franck M.; de Toro-Martín J.; Varin T. V.; Garneau V.; Pilon G.; Roy D.; Couture P.; Couillard C.; Marette A.; Vohl M. C. Gut Microbial Signatures of Distinct Trimethylamine N-Oxide Response to Raspberry Consumption. Nutrients 2022, 14 ( (8), ), 1656 10.3390/nu14081656. 35458219 PMC9027468

[ref54] Sanders M. E.; Klaenhammer T. R. Invited Review. The Scientific Basis of Lactobacillus Acidophilus NCFM Functionality as a Probiotic. J. Dairy Sci. 2001, 84 (2), 319–331. 10.3168/jds.S0022-0302(01)74481-5. 11233016

[ref55] Hsieh P. S.; Ho H. H.; Hsieh S. H.; Kuo Y. W.; Tseng H. Y.; Kao H. F.; Wang J. Y. Lactobacillus Salivarius AP-32 and Lactobacillus Reuteri GL-104 Decrease Glycemic Levels and Attenuate Diabetes-Mediated Liver and Kidney Injury in Db/Db Mice. BMJ Open Diabetes Res. Care 2020, 8 (1), e001028 10.1136/BMJDRC-2019-001028. PMC7202753 32332068

[ref56] Ivona S. L.; Nicolescu A.; Popa S.; Sandu M.; Mota M.; Kovacs E.; Deleanu C. Relationship between Urinary Metabolites and Type 2 Diabetes Mellitus by Proton Nuclear Magnetic Resonance Spectroscopy Method (1H-NMR). Endocrine Abstracts 2016, 41, GP91 10.1530/endoabs.41.GP91.

[ref57] Tam Z. Y.; Ng S. P.; Tan L. Q.; Lin C. H.; Rothenbacher D.; Klenk J.; Boehm B. O.; Kiat K. G. K.; Suwanchaikasem P.; Tipthara P.; Yang S. Y.; Becker T.; Stingl J.; Koenig W.; Riepe M.; Peter R.; Geiger H.; Ludolph A.; Arnim C. V.; Nagel G.; Weinmayr G.; Rapp K.; Denkinger M. D.; Dallmeier D.; Steinacker J. M.; Laszlo R. Metabolite Profiling in Identifying Metabolic Biomarkers in Older People with Late-Onset Type 2 Diabetes Mellitus. Sci. Rep 2017, 7 (1), 1–12. 10.1038/s41598-017-01735-y. 28663594 PMC5491522

[ref58] Hanafy M. M.; Lindeque J. Z.; El-Maraghy S. A.; Abdel-Hamid A. H. Z.; Shahin N. N. Time-Based Investigation of Urinary Metabolic Markers for Type 2 Diabetes: Metabolomics Approach for Diabetes Management. BioFactors 2021, 47 (4), 645–657. 10.1002/biof.1731. 33836111

[ref59] Schicho R.; Shaykhutdinov R.; Ngo J.; Nazyrova A.; Schneider C.; Panaccione R.; Kaplan G. G.; Vogel H. J.; Storr M. Quantitative Metabolomic Profiling of Serum, Plasma, and Urine by 1H NMR Spectroscopy Discriminates between Patients with Inflammatory Bowel Disease and Healthy Individuals. J. Proteome Res. 2012, 11 (6), 3344–3357. 10.1021/pr300139q. 22574726 PMC3558013

[ref60] Jung S.; Lee M.-S.; Chang E.; Kim C.-T. Mulberry (Morus Alba L.) Fruit Extract Ameliorates Inflammation via Regulating MicroRNA-21/132/143 Expression and Increases the Skeletal Muscle Mitochondrial Content and AMPK/SIRT Activities. Antioxidants 2021, Vol. 10, Page 1453 2021, 10 (9), 1453 10.3390/antiox10091453. PMC8468054 34573085

[ref61] Lu L.; Li J.; Yew D. T.; Rudd J. A.; Mak Y. T. Oxidative Stress on the Astrocytes in Culture Derived from a Senescence Accelerated Mouse Strain. Neurochemistry International 2008, 52, 282–289. 10.1016/j.neuint.2007.06.016. 17664019

[ref62] Wang B.; Tang X.; Mao B.; Zhang Q.; Tian F.; Cui S.; Chen W. Anti-Aging Effects and Mechanisms of Anthocyanins and Their Intestinal Microflora Metabolites. Crit. Rev. Food Sci. Nutr. 2022, 0 (0), 1–17. 10.1080/10408398.2022.2123444. 36128763

[ref63] Yang H.; Pang W.; Lu H.; Cheng D.; Yan X.; Cheng Y.; Jiang Y. Comparison of Metabolic Profiling of Cyanidin-3-O-Galactoside and Extracts from Blueberry in Aged Mice. J. Agric. Food Chem. 2011, 59 (5), 2069–2076. 10.1021/jf1033619. 21302942

[ref64] Abu Bakar Sajak A.; Mediani A.; Maulidiani; Ismail A.; Abas F. Metabolite Variation in Lean and Obese Streptozotocin (STZ)-Induced Diabetic Rats via 1H NMR-Based Metabolomics Approach. Appl. Biochem. Biotechnol. 2017, 182 (2), 653–668. 10.1007/s12010-016-2352-9. 27995574

[ref65] Briggs J. P.; Levitt M. F.; Abramson R. G. Renal Excretion of Allantoin in Rats: A Micropuncture and Clearance Study. American Journal of Physiology - Renal Fluid and Electrolyte Physiology 1977, 233 ( (5), ), F373 10.1152/ajprenal.1977.233.5.F373. 920807

[ref66] Chung H.-H.; Lee K. S.; Cheng J.-T. Decrease of Obesity by Allantoin via Imidazoline I 1-Receptor Activation in High Fat Diet-Fed Mice. Evidence-Based Complementary and Alternative Medicine 2013, 2013, 589309 10.1155/2013/589309. 23606885 PMC3626183

