# Supplementary material for: Effect of Acylated and Nonacylated Anthocyanins on Urine Metabolic Profile during the Development of Type 2 Diabetes in Zucker Diabetic Fatty Rats
Source: J Agric Food Chem. 2022 Nov 21;70(48):15143–56. doi: 10.1021/acs.jafc.2c06802 (PMC9732871; doi:10.1021/acs.jafc.2c06802)
Supplement: Supplementary file 1 — jf2c06802_si_001.pdf [file jf2c06802_si_001.pdf]

Effect of acylated and nonacylated anthocyanins on urine metabolic profile during the development of type 2 diabetes in Zucker diabetic fatty rats

Kang Chen[†], Xuetao Wei[‡], Zhang Jian[§], Maaria Kortesniemi[†], Yumei Zhang[§], Baoru Yang[†*]

[†]Food Sciences, Department of Life Technologies, University of Turku, FI-20014 Turun yliopisto, Finland

[‡]Beijing Key Laboratory of Toxicological Research and Risk Assessment for Food Safety, Department of Toxicology, School of Public Health, Beijing University, Beijing 100191, China

[§]Department of Nutrition and Food Hygiene, School of Public Health, Beijing University, Beijing 100191, China

* Author for Correspondence:

Professor Baoru Yang,

baoru.yang@utu.fi; Tel: +358 452737988

Table S1 ^{1}H chemical shift assignments of the metabolites observed in the NMR spectra of urine samples.

Metabolites	Assignments	δ1H (multiplicity)	Bins used for quantification
Unknown		1.14(d)	1.125-1.160
3-Methyl-2-oxovalerate	αCH_3	1.09 (s)	1.08-1.10
Ethanol	CH_3	1.20(t), 3.40(q)	1.170-1.200
Lactate	αCH, βCH_3	1.33(d), 4.10(q)	4.10-4.15
Acetoin	αCH_3, βCH_3	2.21 (s),1.370 (d)	1.37-1.393
Alanine	βCH_3	1.47 (d)	1.472-1.495
Acetate	βCH_3	1.92(s)	1.91-1.94
Succinate	CH_2	2.400(s)	2.400-2.428
2-Oxoglutarate	γCH_2	3.00(t)	2.997-3.035
Citrate	CH_2	2.53(dd)	2.52-2.58
Dimethylamine	CH_3	2.72(s)	2.720-2.730
Trimethylamine	CH_3	2.89(s)	2.885-2.90
Dimethyglycine	CH_3	2.92(s)	2.923-2.935
Creatinine	CH_2	3.02(s)	3.033-3.053
Fumarate	αCH	6.53(s)	6.515-6.540
Allantoin	CH	5.4 (s)	5.39-5.403
Trans-aconitate	CH	6.60 (s), 3.40 (s)	6.590-6.599
Phenylalanine	C2, H, benzene ring;C3, H, benzene ring	7.420(m), 7.30(d)	7.305-7.335

Benzoate	C4, H, benzene ring	7.48(t)	7.47-7.508
Hippurate	C2, C3, C4, C5, C6, H, benzene ring	7.55(t),7.65(t), 7.84(d)	7.53-7.58
Trigonelline	C1, C3, H, pyridine ring	8.85(t), 9.1(s)	8.815-8.865
N-Methylnicotinamide	C2, C6, H, pyridine ring	8.88(d),8.95(d), 9.25(s)	9.265-9.28
Formate	HCOO-	8.46(s)	8.455-8.465
Urea	NH_2	5.78(b)	5.600-5.900
Glucose	αCH	4.68 (d)	4.628–4.636
Arabinose	αCH	4.50 (d)	4.505-4.525
Mannose	αCH	5.2 (d)	5.185-5.195

Table S2 Fold change of the urinary metabolites in M group compared to other groups at week 1 timepoint.

Metabolites	M/Con	M/ND	M/L-NAAB	M/H-NAAB	M/L-AAPP	M/H-AAPP
Acetate	0.76*	0.48***	1.42	1.89	0.89	1.67
Acetoin	1.53**	1.38**	1.25*	1.63**	1.42*	1.76**
Alanine	1.67**	1.64***	1.25*	1.98***	1.95**	1.73**
Allantoin	0.88*	0.86*	0.92	0.99	1.02	0.98
Benzoate	0.85	0.68*	1.09	1.21	1.49*	1.50*
Trans-aconitate	0.92	0.89	1.06	1.22	1.11	0.95
Citrate	1.03	1.10**	1.03	0.93*	0.96	1.07
Creatinine	0.74**	0.70***	1.01	1.01	0.97	0.98
Dimethylamine	1.07	0.82	0.75	0.40**	0.42	0.46
Dimethylglycine	0.78**	0.70**	0.82*	0.82*	0.90	0.83*
Ethanol	0.90	0.50	0.92	1.02	1.04	1.01
Formate	1.15	1.02	0.95	0.73	1.55	0.79
Fumarate	0.74**	0.85	0.91	0.81**	0.75**	0.76**
Hippurate	1.37	1.26	0.97	0.98	1.41	1.14
Unknown	1.42*	1.48**	0.24	1.08	0.90	0.81
3-Methyl-2-oxovalerate	1.12	1.06	1.16	1.30*	1.37*	1.40*
Lactate	1.17	1.40*	1.16*	1.44**	1.50**	1.48**
N-Methylnicotinamide	1.05	1.08*	0.95	0.99	0.99	0.91
2-Oxoglutarate	0.79**	0.78***	0.95	0.82*	0.76**	0.78**
Phenylalanine	1.04	1.05	1.07	1.05	1.08	1.01
Succinate	0.90*	0.80*	1.11	1.21	1.24	1.19
Trigonelline	1.01	1.02	0.93	0.96	0.98	0.87
Trimethylamine	1.24	1.16	2.08	2.01	0.90	1.85
Urea	1.29***	1.28***	1.08	1.13	1.06	1.11
Mannose	0.98	1.08	0.90	1.00	1.15	0.95
Arabinose	1.42	1.08	1.30	1.18	1.35	1.06
Glucose	1.24	1.38	1.24	1.38	1.27	1.06

*p < 0.05, **p < 0.01, and ***p < 0.001 as compared with the M group

Table S3 Fold change of the urinary metabolites in M group compared to other groups at week 4 timepoint.

Metabolites	M/Con	M/ND	M/L-NAAB	M/H-NAAB	M/L-AAPP	M/H-AAPP
Acetate	5.02**	1.71	1.05	1.47	0.44*	1.02
Acetoin	3.11	2.86	1.42	1.25	1.88	2.57
Alanine	1.08	0.42**	0.91	0.91	0.82	0.81
Allantoin	0.73	0.63*	1.23	0.83*	1.04	0.94
Benzoate	1.3	0.27**	0.77	1.01	0.86	0.62
Trans-aconitate	0.39**	4.49**	0.56*	0.60	0.86	0.80
Citrate	0.59**	0.64*	0.76	0.72*	1.24	0.7*
Creatinine	0.41***	0.46***	0.83	0.8	1.14	0.81*
Dimethylamine	0.79	0.91	0.93	0.92	1.34	0.84
Dimethylglycine	0.89	0.53***	1.29	0.88	1.13	0.87
Ethanol	9.29***	18.83***	1.83	1.68	1.39	5.42**
Formate	17.7***	17.7***	1.55	1.58	0.92	4.42**
Fumarate	0.36*	0.37*	0.9	0.69	0.86	0.51*
Hippurate	0.39***	0.49*	0.89	0.58*	0.67	0.35**
Uknown	20.52**	36.12***	5.73*	2.21	5.03	4.48
3-Methyl-2-oxovalerate	0.97	0.91	1.00	0.78*	0.78	0.87
Lactate	2.91*	1.09	1.21	1.38	1.23	1.77*
N-Methylnicotinamide	0.58***	0.71 **	0. 70*	0.79*	1.06	0.79*
2-Oxoglutarate	0.79*	0.99	0.77*	0.71*	1.13	0.73
Phenylalanine	0.66*	0.72*	1	0.6*	0.99	0.65*
Succinate	1.22	0.75*	1.08	1.32	0.53	1.29
Trigonelline	0.56***	0.67**	0.78*	0.75*	1.01	0.78*
Trimethylamine	4.44*	9.38*	0.71	1.19	0.94	1.04
Urea	1.11	0.63**	1.43	1	1.13	1.35
Mannose	38.16***	14.98***	1.32	1.31	1.11	1.60
Arabinose	8.91***	8.23***	1.39	1.08	1.04	1.80*
Glucose	102.67***	320.12***	1.45	1.39	0.99	1.95*

*p < 0.05, **p < 0.01, and ***p < 0.001 as compared with the M group

Table S4 Fold change of the urinary metabolites in M group compared to other groups at week 8 timepoint.

Metabolites	M/Con	M/ND	M/L-NAAB	M/H-NAAB	M/L-AAPP	M/H-AAPP
Acetate	1.15	0.83	0.71	1.25	0.92	1.11
Acetoin	4.47*	4.78*	1.68	0.97	1.17	0.81
Alanine	0.36**	0.28**	0.89	1.07	0.98	1.05
Allantoin	0.44***	0.42***	0.85	0.86	0.87	0.89
Benzoate	0.36*	0.23**	1.12	0.97	1.43	0.85
Tran-aconitate	0.45***	4.89**	0.88	0.84	1.11	0.83
Citrate	0.54**	0.57***	0.64	0.63	1.17	0.77
Creatinine	0.29***	0.33***	0.84	0.85	0.9	0.87
Dimethylamine	0.66***	0.69*	0.64	0.83	0.74	0.86
Dimethylglycine	0.79*	0.6***	0.92	0.81	0.89	0.98
Ethanol	7.46***	9.64***	1.36	1.3	1.27	1.24
Formate	13.1**	5.91**	1.69	1.72	2.11	2.66*
Fumarate	0.77	0.73*	1.28	0.96	0.72	0.96
Hippurate	0.44**	0.36***	0.77	0.69	0.65	0.71
Unknown	50.44***	46.91**	2.15	1.07	1.3	0.99
3-Methyl-2-oxovalerate	0.90	0.74*	1.06	1.09	1.10	1.11
Lactate	1.59*	1.39*	1.3	1.16	1.25	1.11
N-Methylnicotinamide	0.55***	0.62***	0.95	0.93	0.92	0.93
2-Oxoglutarate	0.76	1.27	0.75*	0.71*	0.96	0.79
Phenylalanine	0.46***	0.57**	1.04	0.73	1.05	1.03
Succinate	1.38	1.32	0.92	1.32	1.06	1.28
Trigonelline	0.55***	0.62***	0.92	0.93	0.91	0.94
Trimethylamine	1.56	0.82	1.05	0.9	0.97	0.91
Uridine	0.37*	0.23***	1.14	0.96	1.41	0.81
Urea	0.71*	0.66*	1.09	0.86	0.82	0.92
Mannose	32.77***	23.96***	1.04	1.13	1.06	1.04
Arabinose	13.03***	8.83***	1.11	1.05	1.06	1.06
Glucose	171.99***	58.21***	0.99	1.09	1.01	1.04

*p < 0.05, **p < 0.01, and ***p < 0.001 as compared with the M group

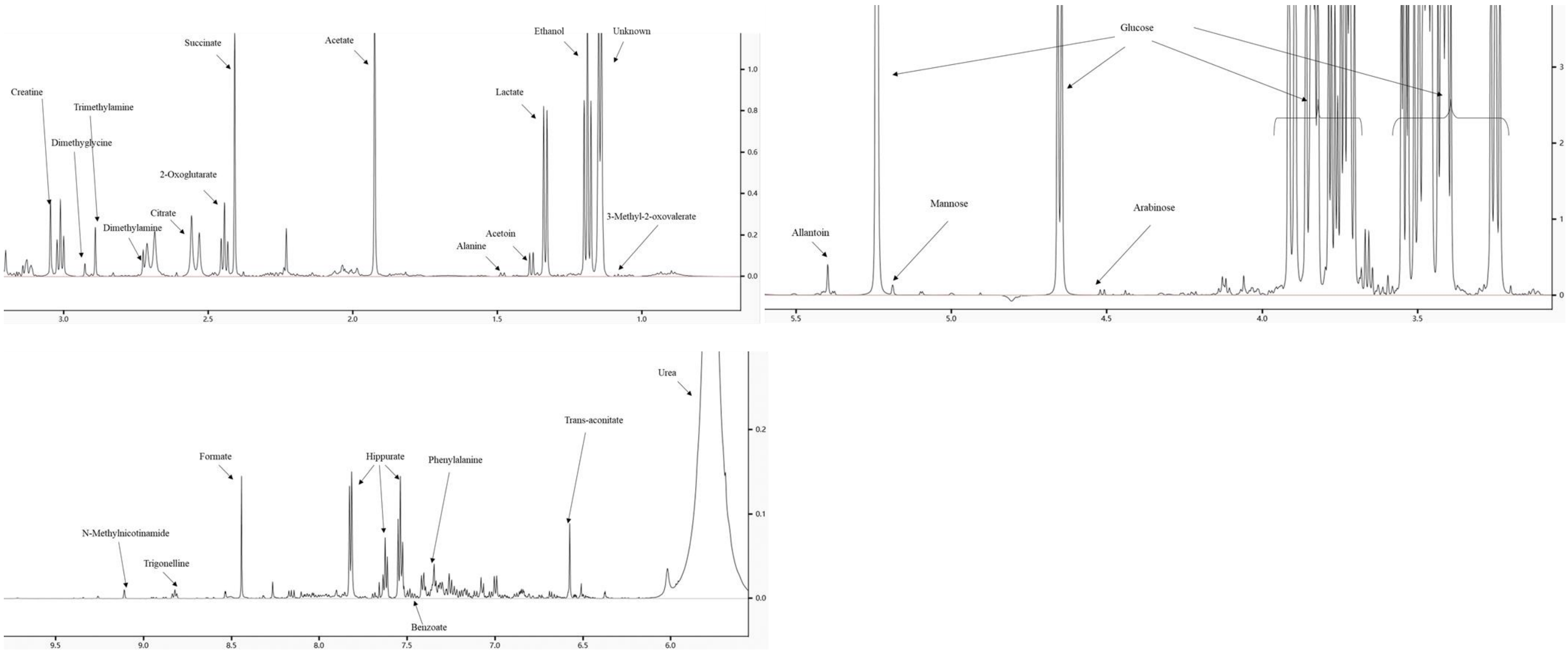

Figure S1 600 MHz *noesypr1d* ^{1}H NMR spectrum of urine sample with identified metabolites

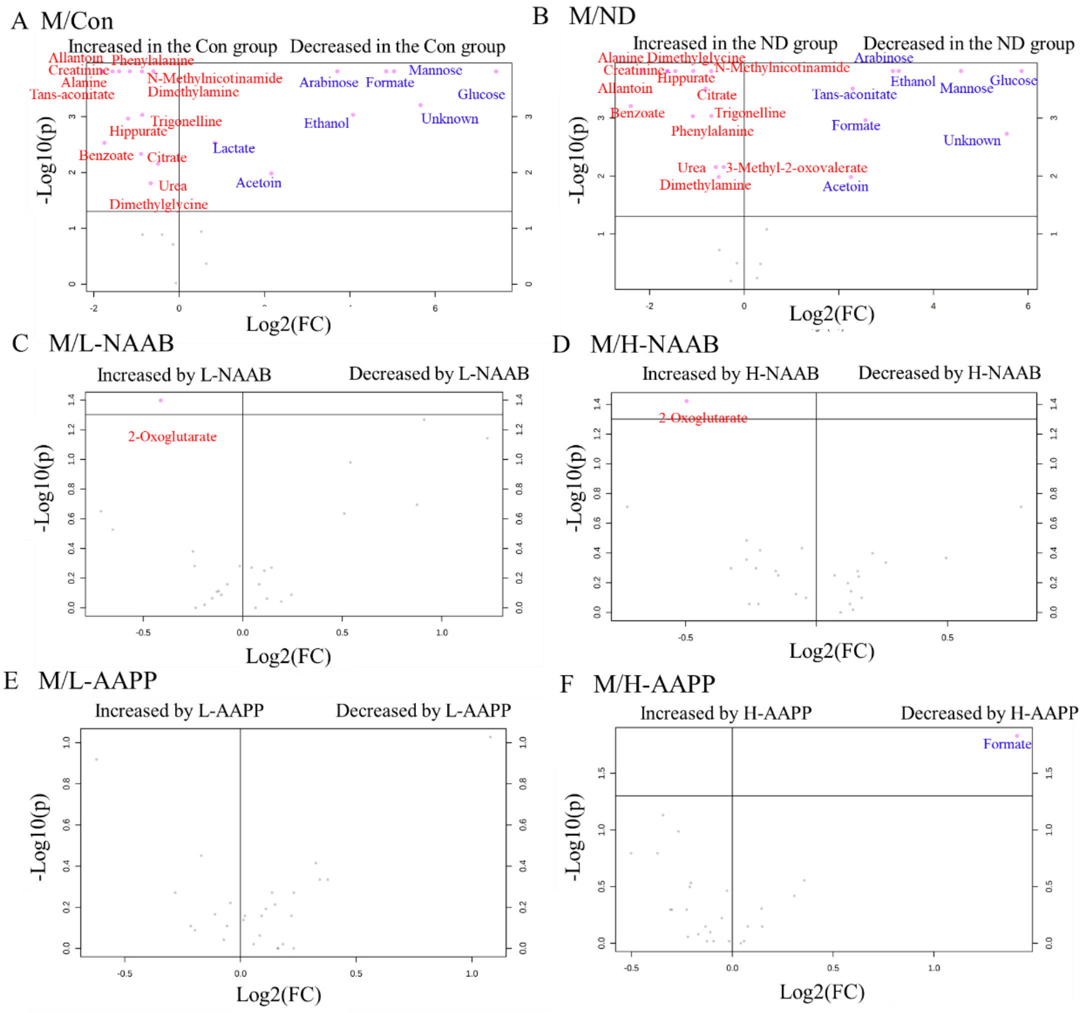

Figure S2 Volcano plots showing the significantly different metabolites from urine at week 8 in M/Con (A), M/ND (B), M/L-NAAB (C), M/H-NAAB (D), M/L-AAPP (E), and M/H-AAPP (F) comparisons. Significance versus log2 fold change is plotted on the y and x axes, respectively.

Figure S3

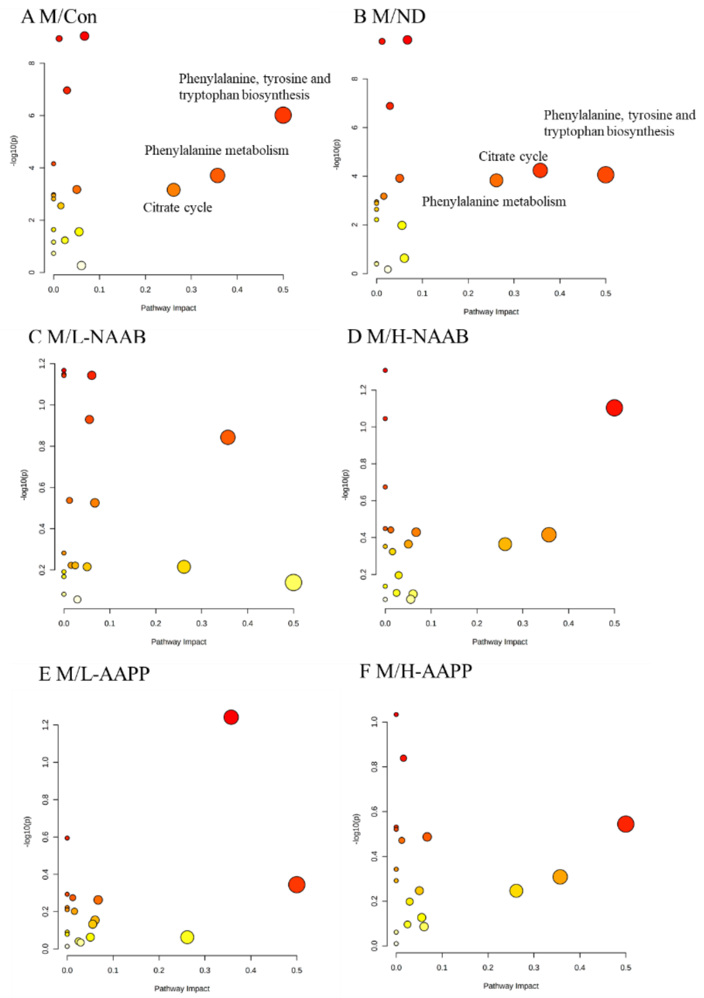

Figure S3 Metabolic pathway analysis generated with the MetaboAnalyst software package based on urine metabolites at week 8, showing altered pathways in M/Con (A), M/ND (B), M/L-NAAB (C), M/H-NAAB (D), M/L-AAPP (E), and M/H-AAPP (F) comparisons. The p-values in the Y-axis are generated from the pathway enrichment analysis, and the X-axis presents the pathway impact values from pathway topology analysis. The node color indicates the p-value from the pathway enrichment analysis (more reddish color indicates more significant changes in the pathway), whereas the node size reflects the pathway impact score. Pathways with small p-values and large pathway impact scores are considered as highly influential. M, ZDF rats fed with high-fat diet; L-NAAB, ZDF rats fed with high-fat diet treated with low dose of nonacylated anthocyanin extract from bilberry; H-NAAB, ZDF rats fed with high-fat diet treated with high dose of nonacylated anthocyanin extract from bilberry; L-AAPP, ZDF rats fed with high-fat diet treated with low dose of acylated anthocyanin extract from purple potato; H-AAPP, ZDF rats fed with high-fat diet treated with high dose of acylated anthocyanin extract from purple potato. Con, lean Zucker rats fed with high-fat diet; ND, lean Zucker rats fed with normal diet.

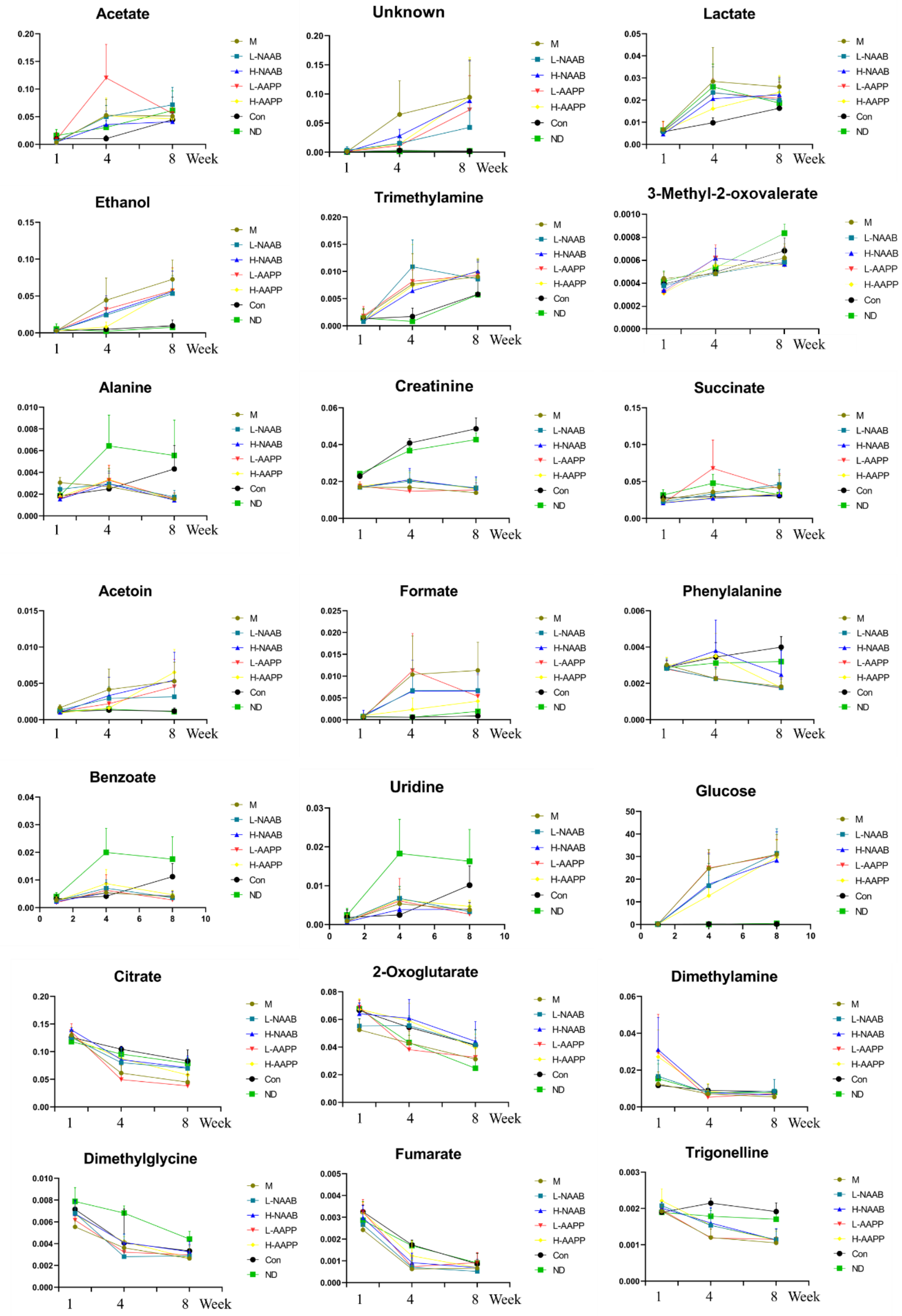
Acetate
Unknown
Lactate
Ethanol
Trimethylamine
3-Methyl-2-oxovalerate
Alanine
Creatinine
Succinate
Acetoin
Formate
Phenylalanine
Benzoate
Uridine
Glucose
Citrate
2-Oxoglutarate
Dimethylamine
Dimethylglycine
Fumarate
Trigonelline
M
L-NAAB
H-NAAB
L-AAPP
H-AAPP
Con
ND
Week

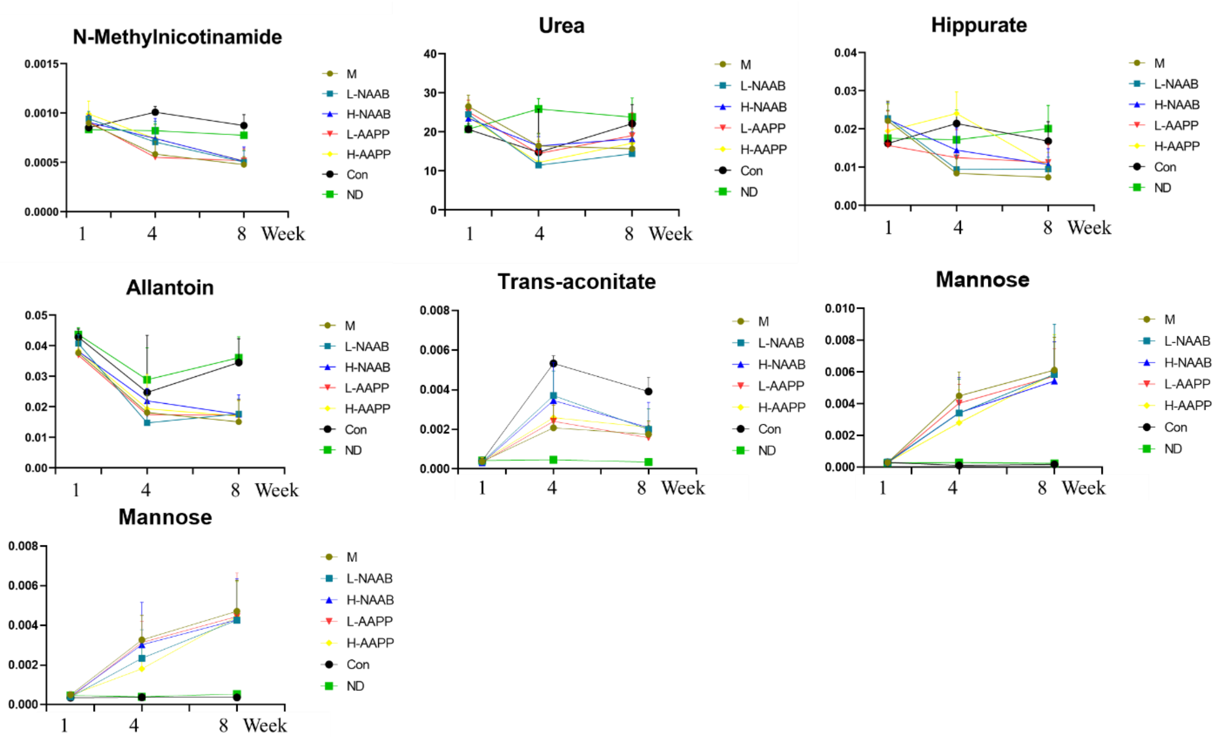

Figure S4 The line charts of metabolites levels at different time points. Y axis indicates binned intensities. X-axis indicates timepoint.
